# Influence of Fiber Diameter and Surface Structure of Polycaprolactone Nanofibrous Materials on Yeast Retention and Biofilm Formation

**DOI:** 10.1021/acsomega.6c07770

**Published:** 2026-10-02

**Authors:** Marta Stindlova, Eva Kuzelova Kostakova, Vera Jencova, Kristyna Havlickova, Vaclav Peroutka, Kamila Zdenkova, Ema Chudobova, Jana Stepanova, David Lukas, Simona Peroutkova

**Affiliations:** † 52735 University of Chemistry and Technology Prague, Faculty of Food and Biochemical Technology, Department of Biochemistry and Microbiology, Technicka 5, 166 28 Prague, Czech Republic; ‡ 496042 Technical University of Liberec, Faculty of Science, Humanities and Education, Department of Chemistry, Studentska 1402/2, 461 17 Liberec, Czech Republic; § Technical University of Liberec, Institute for Nanomaterials, Advanced Technologies and Innovation, Department of Nanochemistry, Studentska 1402/2, 461 17 Liberec, Czech Republic

## Abstract

Understanding how microorganisms interact with nanofibrous materials (NMs) is essential for designing functional surfaces, yet morphology–yeast interactions remain far less studied than bacterial systems. Here, we investigated how fiber diameter and secondary surface structuring of poly-ε-caprolactone (PCL) NMs affect yeast retention, adhesion, biofilm development, and cell transfer. Five PCL NMs were prepared from two PCL molecular weights (PCL45, PCL80) as smooth fibers and as structured fibers generated by two distinct post-processing routes: polymer-spray-induced texturing (PCL45_80, PCL80_80) and enzymatic surface restructuring (PCL80_E). Interactions with model yeasts (*Candida albicans* ATCC 10231 and ATCC 90028; *Saccharomyces cerevisiae* ATCC 9763 and CCM 8381) were assessed by retention, biofilm biomass and metabolic activity during static growth (2 h, 24 h, 72 h), and SEM imaging; cell transfer from colonized agar to NMs was measured using an adapted agar-mesh assay. All tested PCL NMs exhibited high retention (84.7–100%), regardless of fiber diameter or secondary structures. Material-dependent differences were most pronounced during early adhesion, whereas biofilm formed on all NMs and its biomass was strongly strain-specific. Overall, NMs with larger fiber diameter (PCL80 series) supported denser yeast colonization than PCL45, while the secondary structures used here did not confer antifungal activity nor enhance biofilm formation. For cell transfer, PCL45_80 exhibited the highest attachment potential, yet ∼50 % of cells remained on the donor surface. Collectively, these results show that PCL nanofiber morphology modulates yeast–surface interactions primarily by tuning early attachment and colonization capacity.

## Graphical abstract

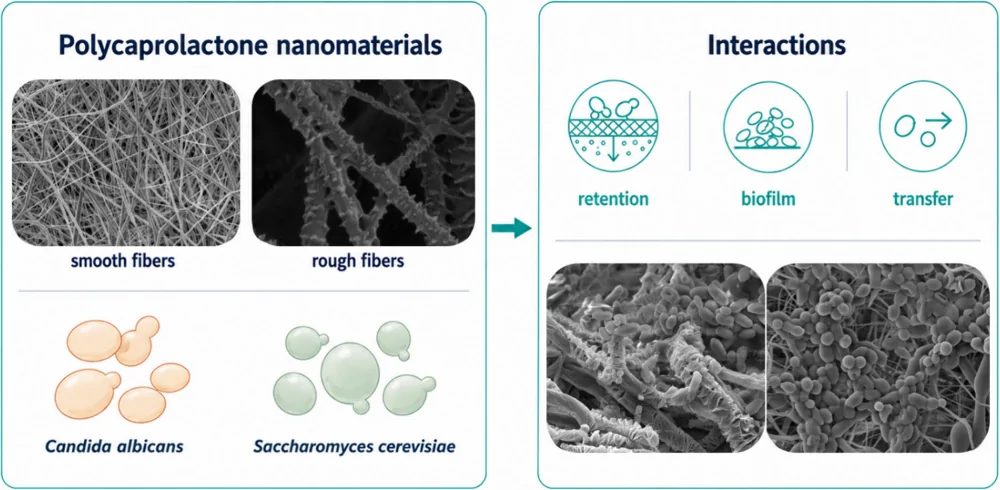

## 1 Introduction

Currently, considerable attention is paid to the research and development of nanofibrous materials (NMs) due to the unique properties of fibers at the nano- and microscales and their wide applications ranging from medicine to storage systems. Among all possible production methods, electrospinning appears to be the most versatile because different polymers can be used, and fiber morphology can be adjusted by production parameters.^1^ Therefore, this method allows the production of smooth, hollow, and/or structured fibers in a single step^2–4^ or follow-up steps.^5–7^

As biocompatible polymers like polycaprolactone (PCL) become components of everyday objects across the population, they inevitably come into contact with unicellular microfungi, i.e., yeasts, either as scaffolds for technological processes or as undesirably colonized surfaces. Most often, studies focus on the antifungal properties of NMs using the native features of electrospun polymers or the addition of antifungals. For example, polyacrylonitrile (PAN) NM was identified as a naturally antifungal material, effectively inhibiting the growth and viability of *Saccharomyces cerevisiae* and *Candida albicans*.^8^ However, PAN is the exception rather than the rule, as most NMs have to be functionalized to exhibit antifungal (or antimicrobial in general) properties. Concerning additional compounds, Firmino *et al.* ^9^ tested the antifungal activity of TiO_2_–CeO_2_ NMs against three species of *Candida* spp. Cerium inhibited biofilm formation in all three species but showed species-specific effect trends depending on cerium concentration. Also, NMs can serve as sophisticated drug delivery systems for commercial antifungals such as clotrimazole^10^ and natamycin^11^ as well as plant extracts.^12^

However, yeasts can develop resistance to antifungal compounds, making the treatment and removal of these yeasts more time-consuming and laborious. Even multidrug-resistant isolates have already been confirmed among *Candida* spp.,^13^ and *C. albicans* has been identified as one of eukaryotic pathogens with increasing resistance.^14^ In 2025, a study in Brazil identified resistance in up to 15% of *Candida* isolates from patients, animals, and environmental samples.^15^ Furthermore, sublethal concentrations of antimicrobials can alter yeast growth and metabolism.^16^

Given growing concern about antifungal resistance and parallels to bacterial resistance spread, new strategies for preventing yeast infections are being sought. Regarding NMs, current research indicates that it is possible to modulate microbe-surface interactions through NM morphology, yet NM–yeast interactions remain largely unexplored. Here, we assess how PCL NM fiber diameter and surface structure affect *C. albicans* and *S. cerevisiae* retention, biofilm formation, and transfer of cells from colonized surfaces. Unique PCL rough fibers were produced by two distinct processes: (i) spraying the electrospun fibers with PCL polymer and (ii) enzymatic fiber degradation. *C. albicans* and *S. cerevisiae* were selected as representative fungal pathogens and model eukaryotes in general, respectively.^8^ Our results suggest that fiber diameter influences yeast biofilm formation and retention more than the secondary structures on the fiber surface created for this study. However, secondary structures can serve as the basis for further modifications to PCL fibers to create even more effective NMs, notably in terms of yeast transfer from various surfaces.

## 2 Material and Methods

### 2.1 Nanomaterials Preparation and Characterization

Poly-*ε*-caprolactone NMs used in this study were prepared by a needleless electrospinning method using a Nanospider NS 1S500U and an AC1100 air conditioner (Elmarco, Czech Republic) and their individual characteristics, spinning conditions and post-processing modifications are described: (i) in Lencova *et al.*:^17^ PCL45 and PCL80 and (ii) Table 1: PCL45_80, PCL80_80 and PCL80_E. The basic polymer material for the formation of polymer solutions was supplied by Merck (Germany) for PCL80 (molecular weight 80,000) and by Corbion (Netherlands) for PCL45 (molecular weight 45,000, Purasorb PC08). Post-processing was performed to enhance the surface structure of the stretched nanofibrous material (PCL45_80 and PCL80_80). A diluted PCL80 solution, prepared at a concentration of 1 wt % in pentyl acetate (PeAc), was sprayed onto both sides of the nanofibrous layer fixed in the frame. After spraying, excess polymer solution was carefully removed from the surface using paper towels. The treated samples were then left to dry under ambient conditions. Another type of post-processing treatment involved short-term enzyme-catalysed degradation and the preparation of a structured material PCL80_E. This NM was prepared by soaking the PCL80 NM in a phosphate-buffered saline (PBS) lipase solution from *Pseudomonas cepacia*, at a concentration of 5 units of enzyme per 10 mg of material. After 24 h at 37 °C with gentle mixing, the materials were removed from the solution, washed in sterile distilled water and left to dry at room temperature. The untreated fibers exhibited a rather smooth surface and a cylindrical morphology, whereas the treated fibers showed pronounced surface roughening and texturing; the formed secondary structures are shown in Figure 1 and Figure 2. Finalized NMs were sterilized by ethylene oxide (12 h, Anprolene sterilizing device AN74, Andersen Sterilizer, UK) according to the ISO 11135-1 “Sterilization of products for health care sterilization with ethylene oxide, part 1: Requirements for the development, validation and continuous control of the sterilization procedure for medical devices” and vented at room temperature in a chemical fume hood for at least 2 weeks to ensure complete removal of residual ethylene oxide. Before use, the NMs were sterilized by UV light for 15 min.

**Table 1:** Tested Nanomaterials with Secondary Structures on Their Fiber Surface^a^

sample	approximate final areal weight (g/m^2^)	solvent system (chloroform: ethanol, w/w)	polymer concentration for smooth fibers (w/w)	post-processing modification
PCL45_80	21.7	8:2	16	both-side application of 1 wt % PCL80 in PeAc; wet areal weight after blotting: 94 ± 11 g/m^2^
PCL80_80	21.5	8:2	10	both-side application of 1 wt % PCL80 in PeAc; wet areal weight after blotting: 137 ± 38 g/m^2^
PCL80_E	17.0	8:2	10	complete wetting in the enzymatic lipase solution (5 U/10 mg material) in PBS for 24 h

a PeAc, penthylacetate; PBS, phosphate buffer saline.

**Figure 1:**
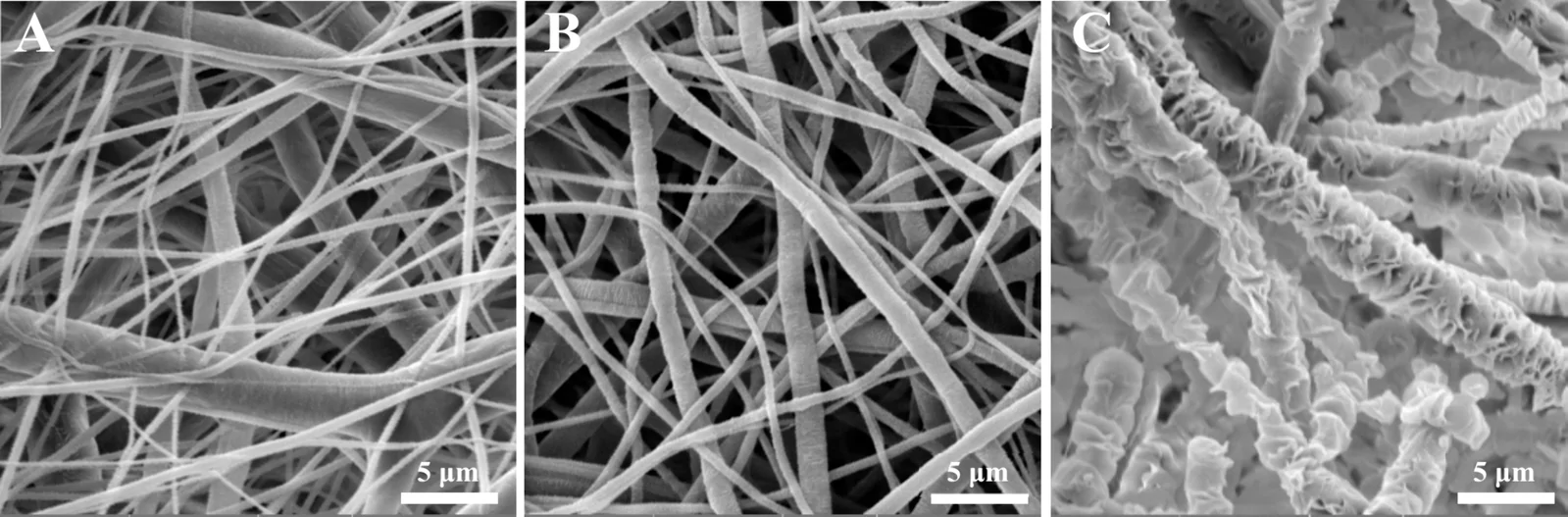
SEM images of secondary structures created on polycaprolactone fibers for this study, according to Table 1, i.e., PCL45_80 (A), PCL80_80 (B), PCL80_E (C).

**Figure 2:**
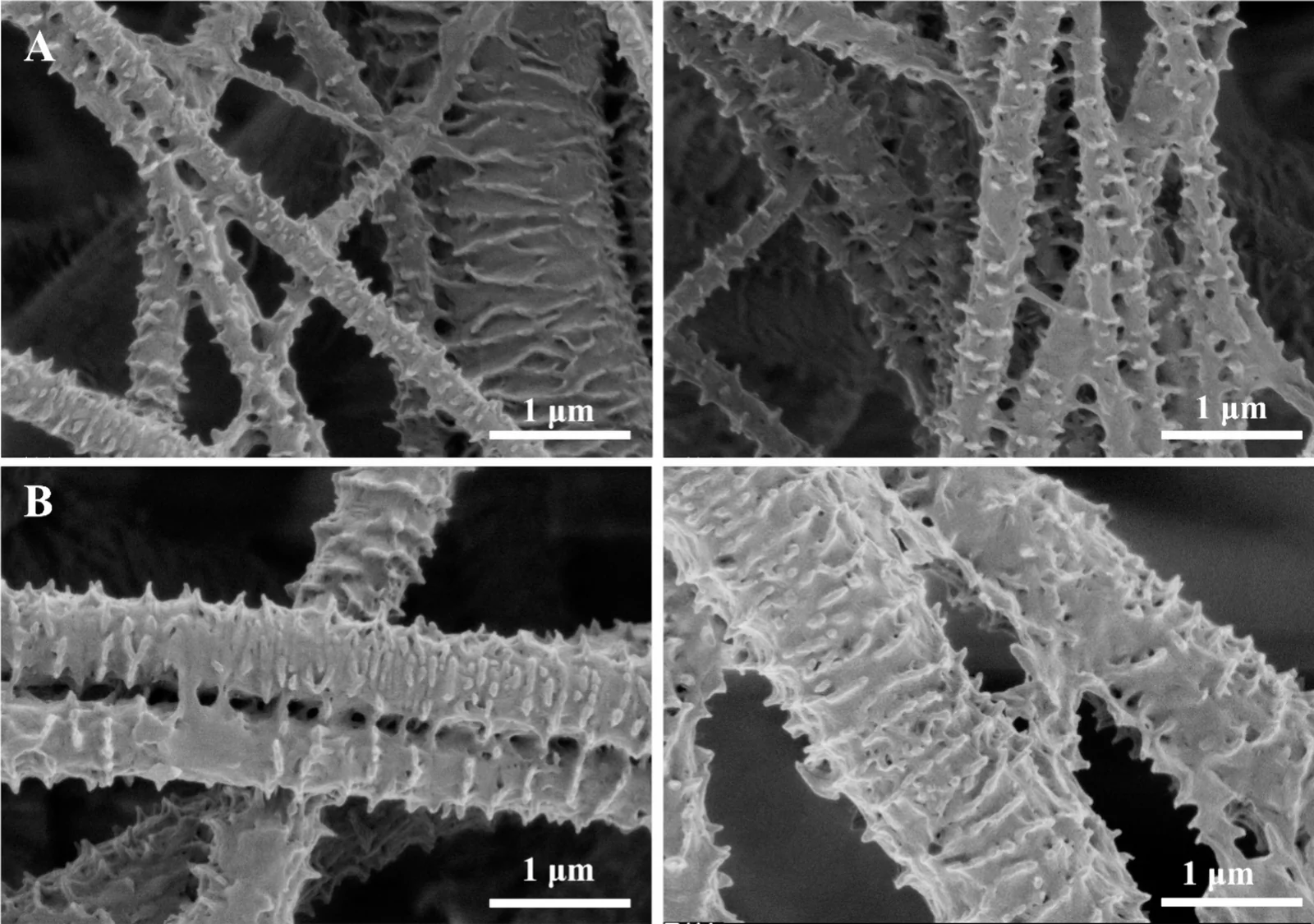
SEM images of secondary structures created on PCL fibers by dilute polymer solution spraying with a detail showing the surface structure of the fibers treated by spraying diluted polymer solutions: PCL45_80 (line A), PCL80_80 (line B).

The structure of the untreated as well as post-process treated rough NMs were observed by scanning electron microscopy (SEM) using Vega S3B (Tescan, Czech Republic) microscope at lower magnifications (see Figure 1 and Figure 3). Detailed surface imaging of the fibers modified by spraying with a diluted polymer solution (PCL45_80 and PCL80_80, see Figure 2) was subsequently performed using a Zeiss ULTRA Plus (Carl Zeiss AG, Germany) scanning electron microscope operated at an accelerating voltage of 1 kV and a beam current of 1–2 pA. Prior to imaging, the samples were coated with a 2 nm platinum layer to provide surface conductivity.

**Figure 3:**
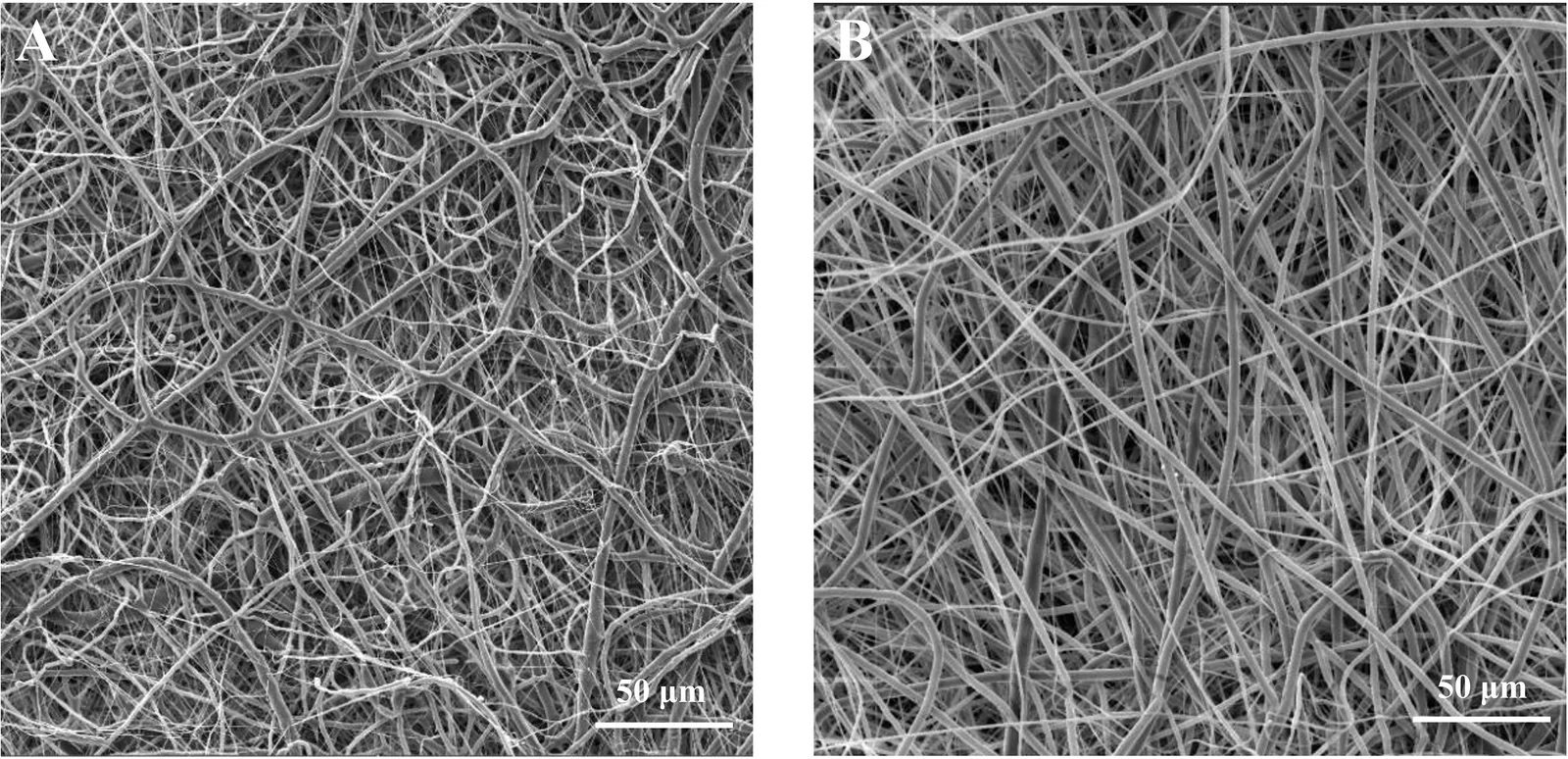
SEM images of larger homogenous areas of NMs PCL45 (A) and PCL80 (B).

The specific surface area of the unmodified NMs and post-process modified NMs by spraying with a diluted polymer solution, namely PCL45, PCL80 and PCL45_80, and PCL80_80, was determined by the Brunauer–Emmett–Teller (BET) method using an Autosorb 6100-XR-XR instrument (Anton Paar, Austria). For each measurement, 0.5–1.0 g of a sample was used. Before analysis, the samples were degassed at 45 °C with a heating rate of 1 °C/min for at least 72 h and until they passed the degassing quality test. Nitrogen served as the adsorbent at 77.35 K. The BET specific surface area was determined by KAOMI software from the adsorption isotherm over the relative pressure range p/p0 = [0.05–0.3]. PCL80_E was not tested using BET analysis.

The chemical compositions of PCL80 and PCL80_E NMs were also investigated using a Fourier transform infrared (FTIR) spectrometer (Nicolet iZ10; Thermo Fisher Scientific, Waltham, MA, USA). The samples were placed on an ATR diamond crystal, and the spectrum analysis was performed in the region within the 4000–400 cm^–1^ range with a spectral resolution of 4 cm^–1^. Atmospheric and baseline corrections were applied.

### 2.2 Yeast Strains and Culture Conditions

Four yeast strains used in this study, namely *Candida albicans* ATCC 10231 (eq CCM 8215), *Candida albicans* ATCC 90028 (eq CCM 8261), *Saccharomyces cerevisiae* ATCC 9763 (eq CCM 8191), and *Saccharomyces cerevisiae* CCM 8381, were obtained from the Czech Collection of Microorganisms (CCM, Brno, Czech Republic). The yeasts were stored in malt extract broth (MEB, Oxoid Ltd., Great Britain) with 25 wt % of glycerol (Penta, Czech Republic) in a freezer (−80°C). For recultivation, the culture was inoculated into sterile MEB and incubated for 48 h at 25 °C. For experiments concerning the enumeration of colony-forming units (CFU), malt extract agar (MEA, Oxoid Ltd., Great Britain) was used, and plates were incubated for 48 h at 25 °C.

### 2.3 Retention Assay

Filtration of individual yeast suspensions was performed as described in Lencova *et al.*.^18^ Briefly, 3 mL of each yeast suspension (optical density 0.5 and 1.0 McFarland, approx. 10^5^ and 10^6^ cells, respectively) were filtered through a sterile PCL membrane (5 cm diameter) into the bottom part of the apparatus. Afterwards, yeast cells in both the filtered suspension and the filtrate were quantified by serial decimal dilution in physiological saline solution (0.9% NaCl) and plotted on MEA. After CFU enumeration, log removal (CFU/mL) and retention rate (%) were calculated. The filter was halved; one half was evaluated by SEM, and the other was halved again and placed on an MEA (either top or bottom up) and incubated for 48 h at 25 °C.

### 2.4 Biofilm Formation

Yeast biofilms were grown from 3 mL of MEB containing approximately 10^5^ CFU/mL in 12-well microtiter plates (TPP, Switzerland) on either the polystyrene (PS) plate bottom or an NM. The 1 cm × 1 cm NMs were sterilized before each analysis by UV radiation (20 min, room temperature) and placed into the suspension. Every experiment included negative sterility controls (medium and NMs). Such prepared microtiter plates were incubated statically at 25 °C for 2, 24, and 72 h.

Preliminary data about adhesion and biofilm formation on PS (to choose the aforementioned time points) were obtained by crystal violet (CV) staining according to the protocol by Djordjevic *et al*.,^19^ i.e., after a specific time, the microtiter plates were washed three times with sterile saline solution, air-dried for 45 min at room temperature, stained with CV for 45 min at room temperature, washed three times with sterile distilled water, air-dried, and observed in an optical microscope.

### 2.5 Determination of Viable Cells by CFU Enumeration

NMs and PS incubated for the time specified above were subjected to the bacterial protocol published by Lencova *et al.*,^18^ with small modifications. In short, NMs and PS were washed in saline solution five times and sonicated for 3 min in 1 mL of MEB in a sonication bath (Polsonic, Poland). Obtained suspensions were decimally diluted and plated on MEA as 20 μL droplets. The plates were then incubated at 25 °C for 48 h, and then the CFU were enumerated.

### 2.6 Determination of Cell Metabolic Activity by MTT Assay

The metabolic activity of yeast cells was determined using the protocol previously published in Stindlova *et al.*.^20^ In short, the NMs and PS with and without biofilms were incubated in MEB, washed in PBS and incubated with 600 μl of MTT (in full name 3-(4,5-dimethylthiazol-2-yl)-2,5-diphenyl tetrazolium bromide; Merck, Germany) solution in PBS with menadione for 2 h at 25 °C to create formazan. The violet formazan crystals were then dissolved in 1 mL of washing solution consisting of sodium dodecyl sulfate, dimethyl sulfoxide and PBS. Absorbance of 100 μl of each sample was measured at 595 nm (Tecan, Switzerland) in a pre-warmed sterile 96-well plate (GAMA Group, Czech Republic).

### 2.7 Agar-Mesh Yeast Cell Transfer

To determine whether yeast cells can transfer from a defined surface to the NMs, the method of Abrigo *et al.* ^21^ was adapted. In brief, 100 μl of a yeast culture (OD = 0.5 McFarland) was spread on MEA and incubated at 25 °C for two distinct times: 2 or 72 h to simulate a newly colonized area or a biofilm coating, respectively. Then, aluminum foil with several 1 cm × 1 cm openings was placed on the agar plates. Subsequently, 1 cm × 1 cm NMs were placed on the openings, and the plates were incubated for 1 h at 25 °C. After incubation, the NMs were subjected to CFU enumeration and SEM analysis. The areas of openings were wiped with sterile swabs, which were then shaken out into sterile saline solution (maximum speed, 1 min), diluted decimally and plotted on MEA. Four openings without the NM cover served as the growth control for swabs (three) and the general growth control (one). The ratio of transferred yeast cells was determined as shown in eq 1.

1 $$\%\;\mathrm{transferred cells}=\frac{\mathrm{CFU on nanomaterial}}{\mathrm{CFU on nanomaterial}+\mathrm{CFU remaining on agar plate}}\times 100$$

### 2.8 Visualization of Cells on Nanomaterials by Scanning Electron Microscopy

Samples of PS and NMs with biofilms were rinsed gently with PBS. Samples with adhered cells and samples after filtration were treated immediately. All samples were fixed with frozen absolute ethanol (99.8%, Penta, Czech Republic) at 4 °C for at least 15 min (PS and agar-transfers were fixed overnight). Samples were then dewatered at room temperature by the increasing concentration of ethanol (concentrations 60.0–99.9%, 10 min per concentration). After drying at room temperature for at least 24 h, the samples were coated with gold (14 nm) and observed using a SEM Tescan Vega3 SB Easy Probe (Tescan, Czechia).

### 2.9 Statistical Analysis

The experiments were performed in at least three biological replicates and three technical replicates. Data sets were subjected to the Shapiro-Wilk test to assess their normal distribution (p > 0.05) and the Levene’s test for homogeneity of variance (p > 0.05). Normally distributed data were subsequently subjected to multiple comparisons by one-way analysis of variance (ANOVA). The difference was assumed to be significant at p < 0.001, p < 0.01, and p < 0.05. If ANOVA yielded statistically significant results, Bonferroni correction and Tukey HSD (Tukey Honest Significant Differences) were calculated to perform multiple pairwise comparisons of groups’ mean. Statistical analysis was performed using the ORIGINPro 2019b program.

## 3 Results and Discussion

### 3.1 Characterization of Nanomaterials

The untreated fibers were electrospun from PCL with two molecular weights and were characterized using the same approach as described in Lencova *et al.*.^17^ SEM analysis confirmed that the morphology of the resulting nano/microfibrous layers is homogeneous, without significant visible defects (Figure 3). The SEM images were complemented by histograms illustrating the diameters of the electrospun fibers (Figure 4), which showed that the PCL80 fibers generally had larger diameters than the PCL45 fibers. For both samples, the fiber diameters exhibited broad, positively skewed distributions. The Shapiro–Wilk, Anderson–Darling, and Lilliefors tests rejected the assumption of normality at α = 0.05. Nevertheless, as fiber diameters have commonly been reported as the mean ± standard deviation in previous studies, these values are also provided here to facilitate comparison. However, the mean was strongly influenced by extreme values in the right tail, and the standard deviation exceeded the mean, resulting in intervals extending to physically meaningless negative values. Therefore, the median and interquartile range were considered the primary descriptive statistics, and the complete diameter distributions are presented as histograms (see Figure 4). According to the AIC and BIC, the best-fitting distributions were the log-logistic distribution for PCL45 and the inverse Gaussian distribution for PCL80. The detailed statistical results were as follows: (i) PCL45 nanofibers: Shapiro–Wilk test: W ≐ 0.586; p ≐ 4.44 × 10^–16^; Anderson–Darling test: p ≐ 0.0005; Lilliefors test: p ≐ 0.001; Loglogistic distribution: AIC ≐ 130.66; BIC ≐ 136.68; (ii) PCL80 nanofibers: Shapiro–Wilk test: W ≐ 0.800; p ≐ 2.51 × 10^–11^; Anderson–Darling test: p ≐ 0.0005; Lilliefors test: p ≐ 0.001; Inverse Gaussian distribution: AIC ≐ 301.83; BIC ≐ 307.85. Because the statistical analysis was intended only as a descriptive comparison, these fitted distributions were not interpreted further. The data were analysed using MATLAB R2025b. The secondary structures on PCL45 and PCL80 fibers were assessed using SEM analysis and are evenly distributed over all visible fibers (see Figure 1 and Figure 2). Spraying treatment is suitable for both PCL45 and PCL80; however, enzymatic degradation causes extensive restructuring (nanostructuring of the fiber surface), fragmentation and, in an advanced stage, complete disintegration of fibers, as we described in our previous work.^22^ Based on these findings, in the present study, the degradation of PCL80 is well controlled. Although the thinnest fibers undergo disintegration (thus, this procedure is not suitable for PCL45), most remain in the form of fibers with a modified surface. Mechanical properties (strength) naturally deteriorate due to weight loss, and some experiments could not be performed for these reasons (e.g. layer rupture may occur). As for the risk of cell detachment, we did not observe this phenomenon during our experiments. Furthermore, multiple factors influence the intrinsic ability of enzymes to degrade nanofibers, including surface charge, fiber crystallinity and temperature. It is also necessary to consider biological degradation after disposal of the said NM.^23^

**Figure 4:**
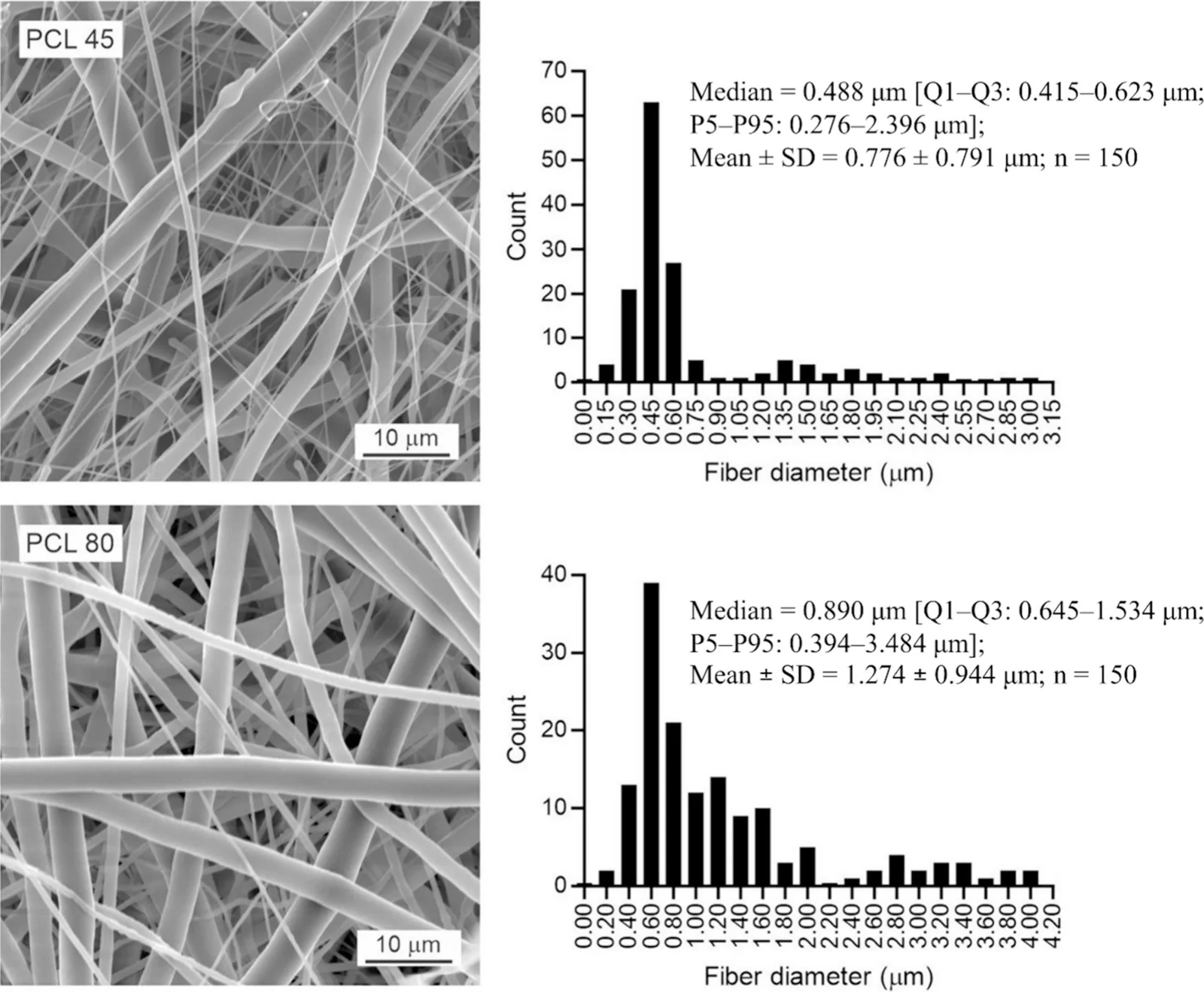
SEM images of the PCL45 and PCL80 NMs supplemented by histograms showing the distribution of the fibers’ diameter.

BET analysis showed that the untreated nanofibrous samples PCL45 and PCL80 exhibited the same specific surface area of 1.8 m^2^/g. After post-process treatment, the specific surface area increased to 2.3 m^2^/g for PCL45_80 and to 3.1 m^2^/g for PCL80_80. These results indicate that the treatment led to an increase in the accessible surface area of the nanofibrous materials with a more pronounced effect observed for the PCL80_80 sample. In comparison with the untreated samples, the post-process treatment increased the specific surface area by approximately 28 % in PCL45_80 and by approximately 72 % in PCL80_80. Concerning PCL80_E, the degassing has been proven to be rather difficult, and therefore, BET analysis could not be performed.

The degradation rate of PCL80_E was expected based on the study by Havlickova *et al.*,^22^ with an average weight loss of 15.2 ± 1.3 % compared to PCL80 after 24 h degradation. Furthermore, during gel permeation chromatography, PCL80_E showed a bulk shift to molecular weights lower than PCL80, which suggests degradation throughout the entire NM. The FTIR analysis of the PCL80_E revealed structural changes primarily evidenced by a significant decrease in absorbance intensity in the region of 1160–1180 cm^–1^, which corresponds to the symmetric stretching vibration of the ester bond (C–O–C) within the polymer backbone (see Figure S1). This marked reduction directly indicates the cleavage of ester linkages and the subsequent breakdown of the macromolecular PCL80 chains. The full spectrum, however, exhibits no accumulation of typical degradation by-products, which are effectively washed away after the degradation procedure.

### 3.2 PCL NMs–Yeast Interactions

Given the relevance of PCL NMs for medical and biotechnological use, we evaluated their interactions with yeasts using a standardized assay set and hypotheses (H) aligned with prior morphology-focused studies with bacteria.^17,18,21,24^ We quantified (i) yeast retention, hypothesizing large retention rate as these cells are larger than bacterial, whose filtered suspensions were in previous studies contained for up to 100.0 %^17^ – H1, (ii) yeast biofilm formation and metabolic activity of biofilm cells, with expected outcomes based on bacterial cell findings on PCL or PA NMs^17,18,24^ − H2 and H3, and (iii) the ability of NMs to transfer yeast cells from colonized surfaces, namely agar plates, inspired by bacterial studies by Abrigo *et al.* ^21^ and Rumbo *et al.* ^24^ which have proven the almost complete removal of bacteria from agar – H4. Therefore, the end points specified below capture the key interactions that determine performance in medical and biotechnological applications, and test the following hypotheses:

H1: NMs retain yeast cells completely during the filtration.
H2: Different yeast strains form different amounts of biofilm on individual NMs at the same time of incubation.
H3: The amount of yeast biofilm/adhered cells on NMs depends on the fiber morphology (smooth or rough surface and/or the diameter).
H4: NMs can transfer a significant number of yeast cells from agar plates.

#### 3.2.1 Retention of Yeast Cells

Generally, NMs are of a porous homogeneous structure, which predetermines them for filtration applications.^25^ The high retention of external particles, both living and non-living, is crucial for both their medical and biotechnological applications. Following our previous research on bacterial cell retention by NMs, where PCL NMs showed up to 100% retention,^17^ we hypothesized that yeast cells would be retained with at least the same efficiency due to their larger size (approx 3.0–10.0 μm in diameter for single cells) compared to previously studied bacteria (approx. 0.5–2.0 μm). Following the previously published studies, we evaluated the impact of both fiber diameter and fiber surface structure. Secondary structures on fibers could affect the viability of cells which would pass through the membrane, therefore lowering the final CFU numbers. But based on Lencova *et al.*,^17^ it is more likely that fiber diameter is the decisive factor. For this experiment, we tested only smooth fibers (PCL45 and PCL80) and rough fibers created by spraying (PCL45_80 and PCL80_80). Material PCL80_E was not suitable for this analysis because it may not withstand filtration conditions, and there were production limitations. Based on these aspects crucial for NMs implementation, PCL80_E is considered unsuitable for filtration applications.

To ensure analysis robustness, suspensions of two different concentrations were filtered through the NMs for all tested yeast strains. Based on the results (retention rates, Table 2, and SEM images, Figure 5), all tested filters retained 84.7 ± 1.6–100.0 ± 0.0 % of yeast cells. The retention rates did not differ significantly (p > 0.05) between the tested NMs of smooth and rough fibers, between different cell concentrations and even between species. Most of the filtrations (75 %) were effective at 95% retention rate or more, and those with lower efficiency (25 % of samples) always appeared at PCL45 or PCL45_80, irrespective of the yeast strain or cell concentration. Therefore, we cannot postulate that the tested NMs can completely remove yeast cells in all cases, thus generally rejecting H1 with a note that, except for PCL45 and its structure variants, tested NMs provided at least 90% retention rate consistently. The decrease in efficiency of PCL45 fibers could be attributed to the material properties of the prepared NMs, mainly as PCL45 has generally thinner fibers than PCL80 (see Figure 4), as in literature, the filtration efficiency has already been shown to be directly dependent on fiber diameter and surface density.^26^

**Table 2:** PCL NMs’ Retention of Yeast Cells Expressed As the Log Enumeration of Retained CFU/mL and the Retention Rate (%)

smooth-fibered material		PCL45				PCL80			
filtered cell concentration		0.5 McFarland		1 McFarland		0.5 McFarland		1 McFarland	
average retention		log_10_(CFU/mL)	%	log_10_(CFU/mL)	%	log_10_(CFU/mL)	%	log_10_(CFU/mL)	%
strain	*C. albicans* 10231	5.0 ± 0.0	99.8 ± 0.1	6.1 ± 0.0	88.9 ± 3.1	5.0 ± 0.0	100.0 ± 0.0	6.2 ± 0.0	100.0 ± 0.0
	*C. albicans* 90028	5.1 ± 0.0	93.1 ± 0.5	6.4 ± 0.0	95.8 ± 0.0	5.1 ± 0.0	99.3 ± 0.3	6.4 ± 0.0	99.9 ± 0.0
	*S. cerevisiae* 9763	5.0 ± 0.0	99.2 ± 0.8	6.3 ± 0.0	98.3 ± 0.1	5.0 ± 0.0	100.0 ± 0.0	6.3 ± 0.0	100.0 ± 0.0
	*S. cerevisiae* 8381	4.9 ± 0.0	85.8 ± 2.1	6.2 ± 0.0	99.2 ± 0.2	4.9 ± 0.0	100.0 ± 0.0	6.2 ± 0.0	99.9 ± 0.1

rough-fibered material		PCL45_80				PCL80_80			
filtered cell concentration		0.5 McFarland		1 McFarland		0.5 McFarland		1 McFarland	
average retention		log_10_(CFU/mL)	%	log_10_(CFU/mL)	%	log_10_(CFU/mL)	%	log_10_(CFU/mL)	%
**strain**	*C. albicans* 10231	5.0 ± 0.0	96.0 ± 1.2	6.2 ± 0.0	98.7 ± 0.2	5.0 ± 0.0	100.0 ± 0.0	6.2 ± 0.0	98.7 ± 0.6
	*C. albicans* 90028	5.1 ± 0.0	84.7 ± 1.6	6.4 ± 0.0	99.1 ± 0.3	5.1 ± 0.0	97.8 ± 0.2	6.4 ± 0.0	99.1 ± 0.6
	*S. cerevisiae* 9763	5.0 ± 0.0	99.4 ± 0.1	6.3 ± 0.0	99.9 ± 0.0	5.0 ± 0.0	99.3 ± 0.1	6.3 ± 0.0	100.0 ± 0.0
	*S. cerevisiae* 8381	4.9 ± 0.0	99.6 ± 0.0	6.2 ± 0.0	95.8 ± 3.0	4.9 ± 0.0	100.0 ± 0.0	6.2 ± 0.0	100.0 ± 0.0

**Figure 5:**
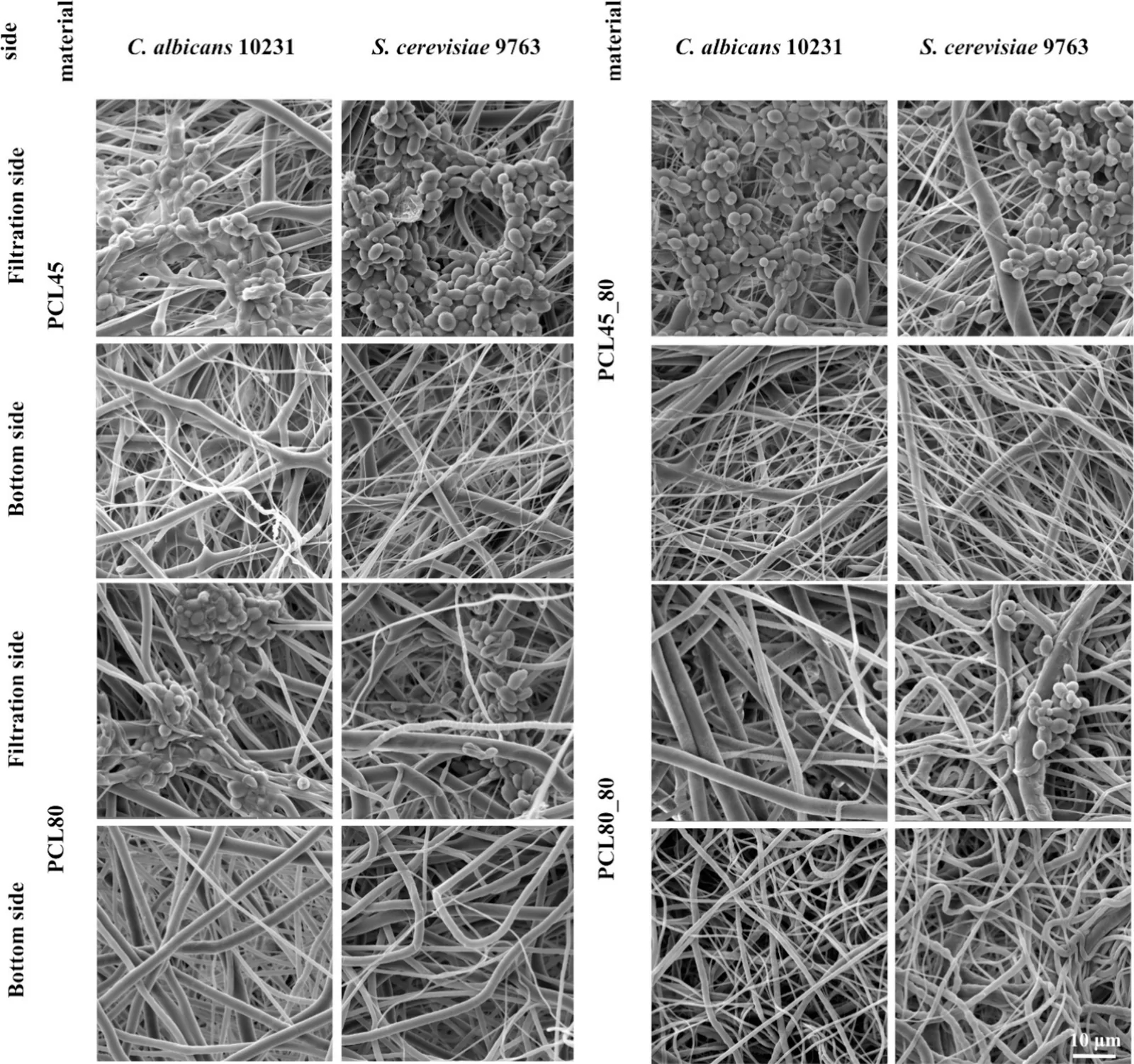
PCL NMs after filtration of *C. albicans* 10231 and *S. cerevisiae* 9763 of OD = 1 McFarland. For each sample, the filtration side (through which the suspension was filtered) and the bottom side are displayed.

Our results correlate well with the bacterial data obtained in the study of Lencova *et al*.,^17^ where PCL45 better retained smaller bacterial cells than PCL80. Bigger yeast cells are more readily retained on all PCL NMs, especially when they cluster, as shown on SEM images in Figure 5. Some SEM images, irrespective of species, also showed remnants of what appeared to be extracellular matrix, based on SEM images of biofilm-forming *S. cerevisiae* published by Zara *et al*.^27^ Our tested yeast cells more easily cluster on the thin fibers with uneven dispersion over the PCL NMs. These images also demonstrate that during filtration, yeast cells can penetrate the larger pores between certain fibers and become trapped there. No cells were visible on the bottom side of the filter membranes.

To further verify retention efficiency, PCL NMs after filtration were incubated on MEA at 25 °C for 48 h and showed that yeast cells grew only on the filtration side, not on the bottom side, and they uniformly covered the whole filter, irrespective of the secondary structures on rough fibers (data not shown). In literature, another polymer, polyamide (PA), has also been tested for yeast retention, and it can lead to a significant reduction in *S. cerevisiae* turbidity in water or beer (99.9 ± 0.1 % and 99.6 ± 2.6 %, respectively) with the starting concentration of 2.9 × 10^8^ CFU/mL.^28^ A more recent study by Mousavi *et al.* ^29^ has focused on nanofiber sponges from PA for filtration. The team showed that smaller pores tend to retain yeast cells better for smaller volumes and bigger pores for higher ones, and that thicker filters achieve higher efficiency. Since only one areal weight of PCL NMs and a single specific volume were tested in our study, it could be an interesting follow-up study to see whether PCL can achieve results similar to PA.

Nevertheless, to the best of the authors’ knowledge, our study is the first to assess how secondary structures on individual fibers in NMs influence the filtration ability of NMs and the filtration capacity of yeast cells. Our results prove that although the structures studied here do not possess any specific antifungal features, they alter the manipulation with PCL NMs and render them less readily wettable by water-based solutions, making the filtration process last longer and favor certain parts of the filter surface.

#### 3.2.2 Biofilm Formation

Yeast biofilm assays were performed on all five PCL NMs to determine how the NM morphology affects biofilm maturation stages. First, the most suitable time points for the biofilm formation were selected. Adherent cells and biofilms on PS were stained with CV at 2 h, 4 h, 6 h, 8 h, 12 h, 24 h, 48 h, 72 h, and 96 h (see Figures S2 and S3). On the basis of the observations, times 2 h (adhesion), 24 h (beginning of biofilm formation; *C. albicans* started to create pseudohyphae), and 72 h (mature biofilm) were chosen for qualitative and quantitative assessment. Biofilm samples were evaluated for CFU/cm^2^ (Figure 6) and metabolic activity using the MTT assay (Figure 7), and selected samples (*C. albicans* 10231 biofilm formation at 24 h and 72 h; other strains yielded similar images and are shown in Figures S4 and S5) were visualized by SEM (see Figure 8).

**Figure 6:**
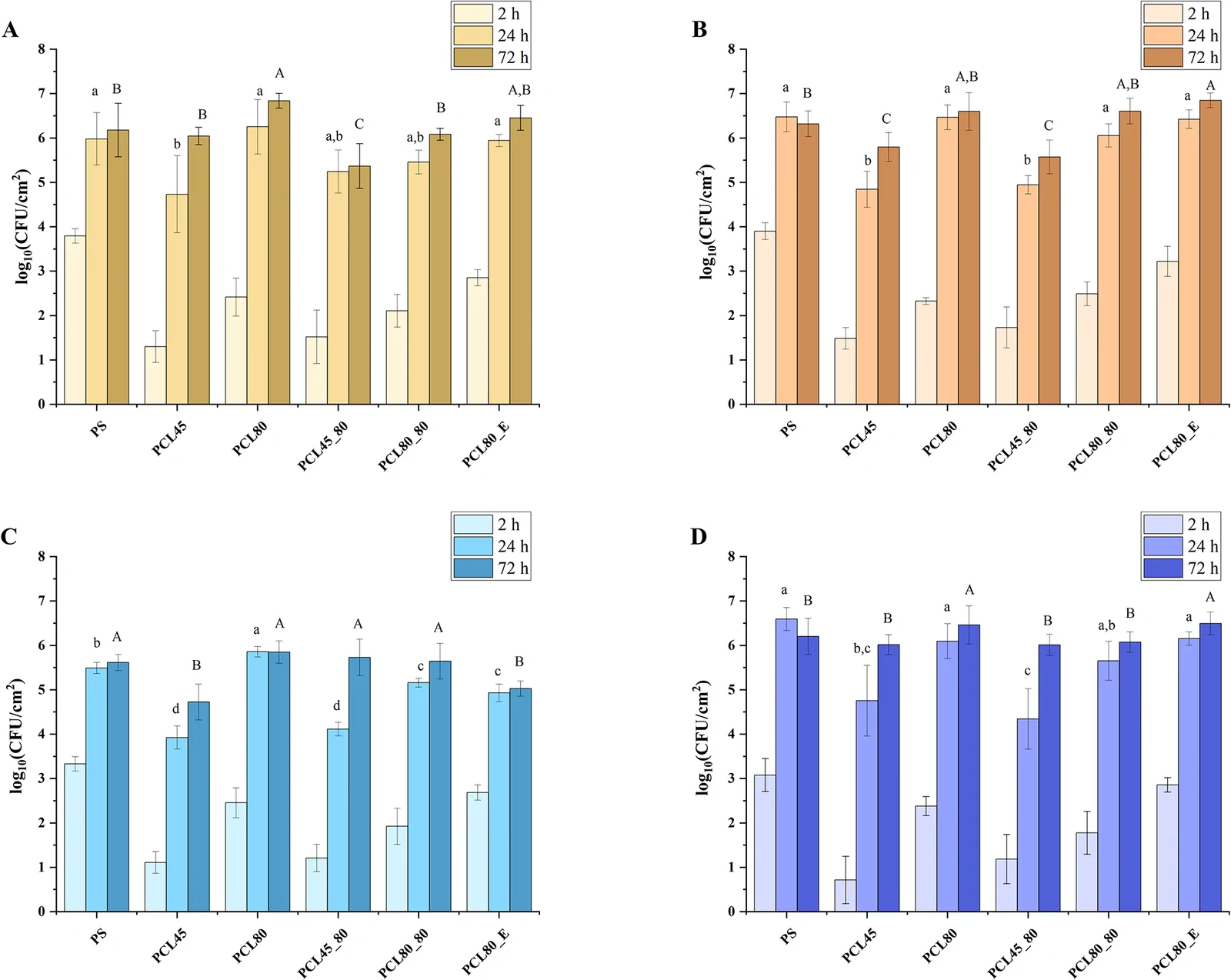
Biofilm formation per cm^2^ at 2 h, 24 h, and 72 h by (A) *C. albicans* 10231, (B) *C. albicans* 90028, (C) *S. cerevisiae* 9763 and (D) *S. cerevisiae* 8381 on reference (PS bottom of a microtiter plate) and PCL NMs. Means that do not share a letter or symbol within the same yeast strain are statistically significant at p < 0.05 (ANOVA and Tukey’s test with Bonferroni correction).

**Figure 7:**
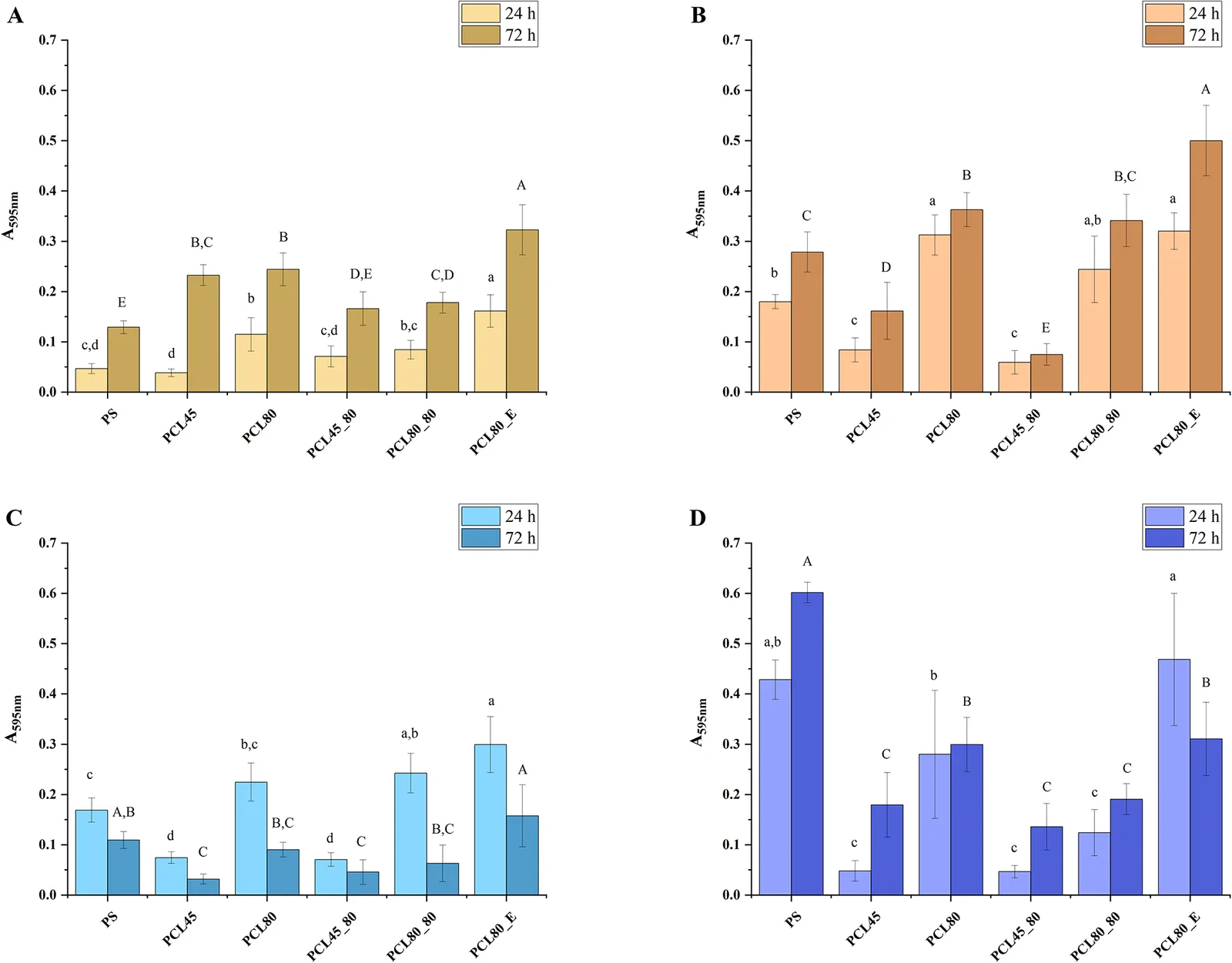
Metabolic activity per cm^2^ of biofilms at 24 h and 72 h created by (A) *C. albicans* 10231, (B) *C. albicans* 90028, (C) *S. cerevisiae* 9763 and (D) *S. cerevisiae* 8381 on reference (PS bottom of a microtiter plate) and PCL NMs. Means that do not share a letter or symbol within the same yeast strain are statistically significant at p < 0.05 (ANOVA and Tukey’s test with Bonferroni correction).

**Figure 8:**
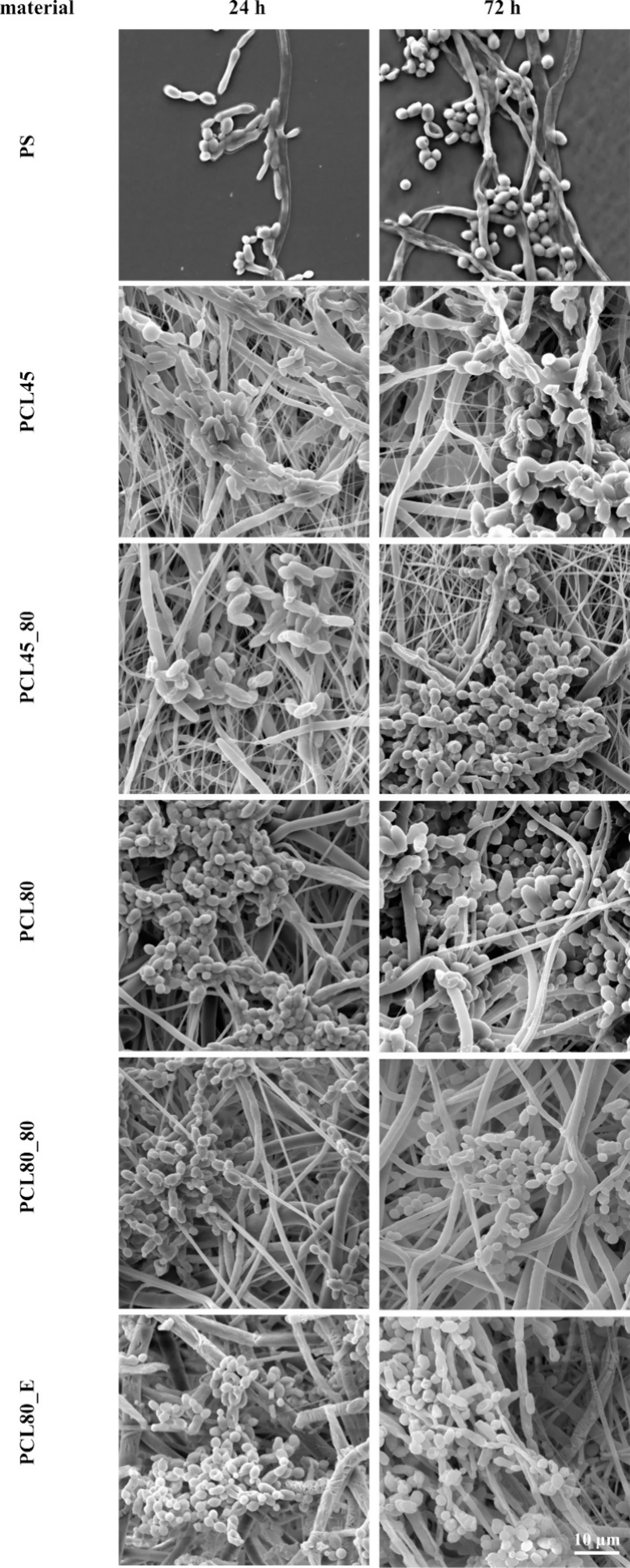
SEM images of biofilms created by *C. albicans* 10231 on all tested materials (PS and PCL NMs) at selected time points of biofilm formation (i.e., 24 h and 72 h).

Regarding biofilm formation dynamics (according to CFU/cm^2^ results), all strains exhibited similar trends of the fastest adhesion to PS and lower numbers for PCL45 and PCL45_80 than PCL80 NMs. Both strains of either *C. albicans* or *S. cerevisiae* colonized all tested materials similarly within the chosen time points. For more details, consult Figure 6 and the text below. Metabolic activity varied much more between each strain, with *C. albicans* having higher activity at 72 h, while *S. cerevisiae* 9763 had a reversed trend, and *S. cerevisiae* 8381 depended on the tested material (see Figure 7). Concerning the ease of manipulation, there were differences between the types of NMs because PCL80_E was almost immediately completely wetted after contact with medium, while PCL45 and PCL80 took somewhat longer (a few minutes) and PCL45_80 and PCL80_80 took the longest (several minutes of actively keeping submerged) to get wet.

The initial adhesion after 2 h incubation showed significant differences between materials, with PS being the most easily colonized material. Considering PS, the *C. albicans* CFU count lay between 3.5 – 4.1 log_10_(CFU/cm^2^) and, for *S. cerevisiae*, it was between 2.5–3.6 log_10_(CFU/cm^2^). PCL45 and PCL45_80 were less colonized than PCL80 NMs, and PCL80_E was considered the most effectively colonized NM after 2 h. MTT and SEM data are not presented for this time point, as these analyses were effectively performed only after 24 h and 72 h. In preliminary experiments, the 2 h end point yielded low and spatially heterogeneous surface coverage, placing the MTT signal close to the assay detection limit and increasing relative variability. Early adhesion is also highly sensitive to initial conditions (e.g., inoculum density, sedimentation, wetting dynamics, and local fiber microtopography), so minor procedural differences can lead to disproportionate apparent effects; over longer incubation, these differences diminish, making 24 and 72 h more robust for analysis. Similarly, for SEM, the low number of attached cells reduces representativeness across fields of view and increases the impact of sample-processing artefacts (fixation or dehydration-induced detachment).

The early biofilms at 24 h showed that the number of CFU/cm^2^ started to become similar over all the tested materials, but PCL45 NMs still had lower CFU/cm^2^ counts than PCL80 NMs, albeit not always significantly. In terms of NMs, *C. albicans* produced biofilms between 3.9–7.0 log_10_(CFU/cm^2^), lowest on PCL45 and highest on PCL80. *S. cerevisiae* showed similar counts and trends, having 3.5–6.8 log_10_(CFU/cm^2^). It is noteworthy that strain *S. cerevisiae* 9763 had the lowest CFU/cm^2^ count of all tested strains and also differed significantly on PS and PCL80_E compared to the other strain *S. cerevisiae* 8381 (p < 0.05). These results hint at differences in the individual yeast biofilm-forming ability on these materials. One reason may be genetic, given the number of repeats in the *FLO11* gene, whose transcription responds to environmental stimuli and activates multiple signalling pathways related not only to biofilm formation but also to metabolism and energy.^27^ At this time point, most rough-fibered NMs did not show any significant differences in colonization and biofilm formation compared to their smooth-fibered counterparts. Metabolic activity at 24 h was generally the lowest for *C. albicans* 10231, reaching only up to the value of 0.16 ± 0.03 for PCL80_E. Except for PS, the metabolic activity of all strains followed the trend set by CFU counts, i.e., PCL45 NMs had lower activity than PCL80 NMs. Strain *C. albicans* 90028 had higher relative metabolic activity, with PCL45 NMs having the lowest values of A < 0.1. Metabolic activity of cells on PCL80 NMs was the highest, but no significant differences were observed between smooth and rough fibers (p > 0.05). *S. cerevisiae* 9763 was the only strain with higher metabolic activity of early biofilm biomass (24 h) than mature biofilms (72 h). *S. cerevisiae* 8381 had the highest deviations between individual biofilm formation on NMs, and its metabolic activity on PS was on par with that on PCL80_E, which usually exhibited the highest activity. SEM images (Figure 8) show the preferential colonization of the surface of fibers, with the possibility of getting to the inner structure of NMs if the pore size allows it. *C. albicans* pseudohyphae were detected.

Mature biofilms at 72 h often showed a significant increase in mean viable biomass (CFU/cm^2^), mainly for PCL45 and PCL45_80. The only exception was *S. cerevisiae* 9763 on PCL80, where the mean seemed to decrease, albeit not significantly. Strain *S. cerevisiae* 9763 still showed the lowest growth and biofilm formation on the tested materials (PS, PCL80 and PCL80_E; p > 0.05). Both the lowest and highest amounts of *Candida* biofilm were found in *C. albicans* 10231; it reached only 5.4 ± 0.5 log_10_(CFU/cm^2^) on PCL45_80 and up to 6.8 ± 0.2 log_10_(CFU/cm^2^) on PCL80. Except for PCL80_80, which significantly increased biofilm formation by *C. albicans* 90028 compared to *C. albicans* 10231 (p < 0.01), there were no significant differences between *Candida* strains (p > 0.05). For *S. cerevisiae*, the lowest determined colonization appeared on PCL45 (3.9 log_10_(CFU/cm^2^) for *S. cerevisiae* 9763) while the highest was observed on PCL80 (6.9 log_10_(CFU/cm^2^) for *S. cerevisiae* 8381). These results also hint at the diameter of fibers being more important for biofilm formation on NMs than secondary structures on the surface of fibers. Although, for example, *C. albicans* 10231 biofilm was reduced compared to a reference (PS) by PCL80 and PCL80_80 by 0.8 log_10_(CFU/cm^2^), it was not the case for *C. albicans* 90028 (similar mean values), which means that rough fibers do not inhibit yeast proliferation, and the biofilm formation is still strain specific. These are important findings concerning the research and development of rough fibers with similar properties to those studied in our experiments. In terms of metabolic activity, *C. albicans* strains usually exhibited a significant increase in the tested materials, most notably in the case of PCL80_E. As mentioned before, *S. cerevisiae* 9763 diminished its metabolic activity over time but remained the most active on PCL80_E. *S. cerevisiae* 8381 increased the metabolic activity with the increase of the log_10_(CFU/cm^2^), albeit not always significantly. The only reduction appeared on PCL80_E by an average of 0.2/cm^2^. SEM images (Figure 8, Figure S5) show generally increased growth of yeasts and more *Candida* pseudohyphae and unicellular growth around them. However, pseudohyphae were not always easily recognized among the fibers of PCL NMs.

To summarize biofilm assay results, the observed morphology-dependent effects were most pronounced during the early adhesion phase, consistent with the established view that fungal biofilm development is a stepwise process in which initial attachment is highly sensitive to substrate physicochemistry and micro-/nano-topography. In *C. albicans*, biofilm maturation is tightly coupled to the yeast-to-hypha transition, and hyphal/pseudohyphal elements act as a key structural scaffold; therefore, material-driven differences may diminish between 24–72 h as filamentation and extracellular matrix increasingly dominate the architecture. This interpretation aligns with studies showing that engineered surface topographies can strongly modulate *C. albicans* attachment and biofilm formation in a feature-size–dependent manner, often with the largest effects at early time points.^30^ In our experiments, larger fiber diameter generally supported higher early colonization than thinner fibers, which is plausibly explained by changes in effective contact area and local sheltering of cells within fibrous networks during attachment.^31^ Divergence between CFU-based biomass and MTT trends is compatible with well-described physiological heterogeneity in mature biofilms (metabolic stratification and mixed morphotypes), meaning higher viable counts do not necessarily translate to proportionally higher metabolic activity.^31^ Furthermore, the tested rough fibers did not prove any anti-yeast or yeast-biofilm-enhancing properties, taking into account that bactericidal properties mean the reduction of a total count of 3 log_10_(CFU) or higher.^32^ Moreover, *C. albicans* did not seem to produce any more or fewer pseudohyphae due to the secondary structures created either by spraying or enzymatic degradation.

Therefore, we can confirm H2 that “Different yeast strains form different amounts of biofilm on individual NMs at the same time of incubation.” and also the biofilm morphology differs greatly. Concerning H3: “The amount of yeast biofilm/adhered cells on NMs depends on the fiber morphology (smooth or rough surface and/or the diameter).”, it is possible to confirm the aspect of fiber diameter because the PCL45 type is usually less colonized than the PCL80 type at all three time points, although not significantly in all cases. Also, due to the surface area, PCL80_E seems to be the fastest-colonized NM even though the amount of viable biofilm cells after 24 h and 72 h is often comparable to PCL80 (except in the case of *S. cerevisiae* 9763, where PCL80 promotes biofilm growth). Metabolic activity is not related to either fiber diameter or secondary structures and has to be strain-specific and time-dependent. However, due to some cases, it could be estimated that yeasts are more active on the PCL80 type, with the highest activity reported for PCL80_E. These estimations show that secondary structures on fibers, notably bigger enzymatic ridges, could be used to increase the metabolism of formed biofilms. Nevertheless, this assumption was not primarily tested in this study and requires follow-up research.

#### 3.2.3 Ability to Detach Cells from a Surface

To complement the retention and biofilm assays, we quantified yeast transfer from a precolonized surface onto NMs because NMs may act as scaffolds that capture and redistribute microbial cells. This end point is relevant for both biomedical risk assessment (surface-to-surface transfer) and biotechnological applications where intentional immobilization is desired. Transfer efficiency is expected to depend strongly on micro/nano-scale architecture and available attachment points, making it a sensitive readout of morphology-driven interactions.

Abrigo *et al.* ^21^ reported that nearly all microbial cells can be transferred from confluent agar biofilms to NMs, and in bacteria, this process depends on fiber diameter relative to cell size and shape. We therefore hypothesize that yeast will transfer less efficiently, because yeast cells are approximately ten times larger than the fiber diameters in our NM set, providing fewer effective attachment points. This hypothesis (H4) was proven correct for both selected time points (2 h and 72 h) because, even though all tested NMs were coated by yeast cells after 1 h at 25 °C (see Figure 11), about half of the CFU remained on the agar plate (see Figure 9 and Figure 10). After 2h-long preincubation (Figures 9A, 9C, 10A and 10C), there were hardly any significant differences for all combinations of tested NMs and strains (p > 0.05). Of the notable differences, *S. cerevisiae* 9763 showed a lower attachment rate to PCL80_E compared to PCL80 (p < 0.05), and the lowest attachment rate of all strains to PCL45_80 (only 34.0 %), see Figure 10A. Because the total CFU count was limited to 3.0 × 10^3^ CFU/cm^2^, it was immensely difficult to localize cells during SEM analysis (data not shown).

**Figure 9:**
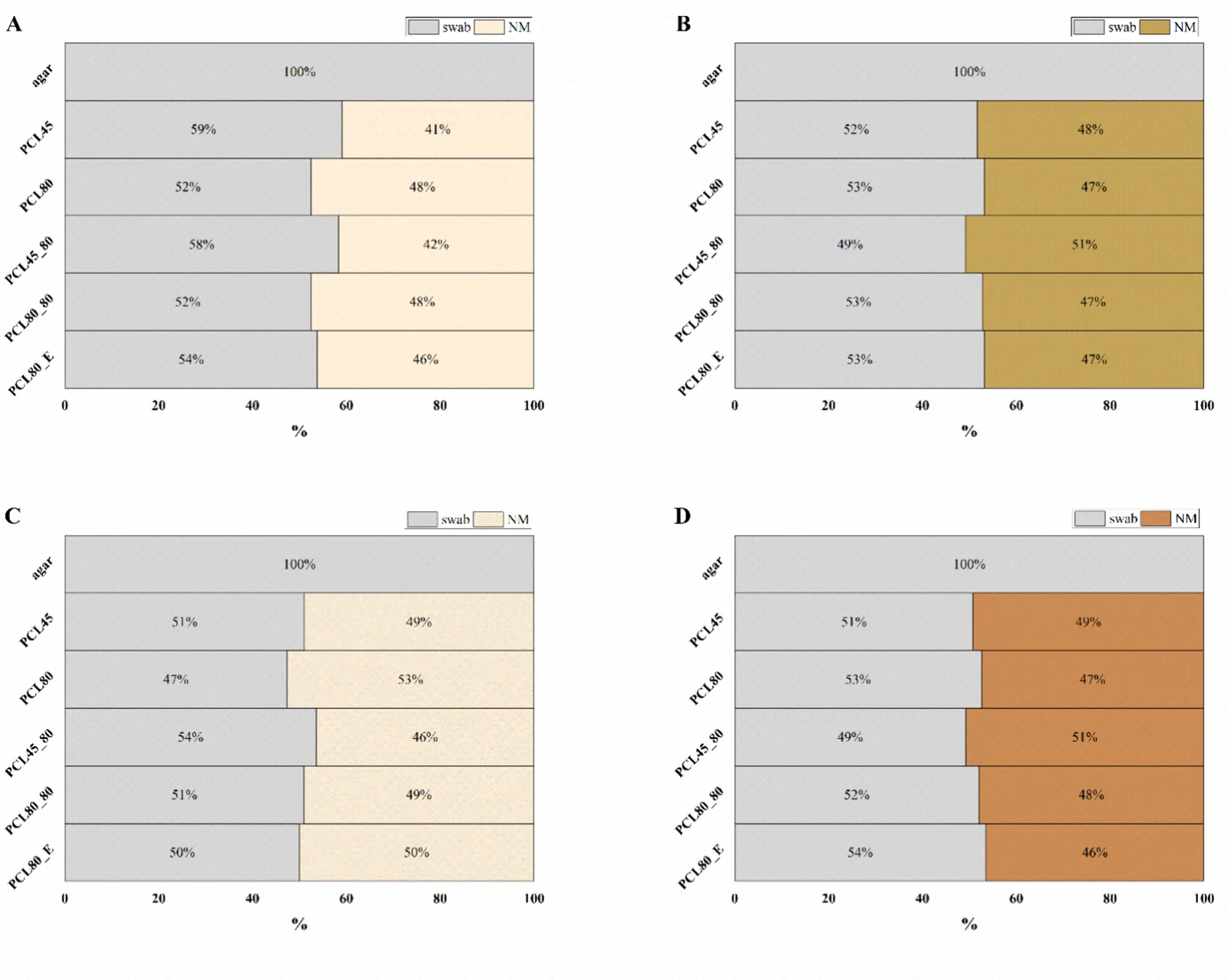
Ratio of agar-NM transfer for *C. albicans* 10231 (A, B) and *C. albicans* 90028 (C, D) for 2 h-long preincubation (A, C) and 72 h-long preincubation (B, D). Every NM transfer is relative to the total CFU count (CFU gained from agar on a swab and CFU gained from NM sonication).

**Figure 10:**
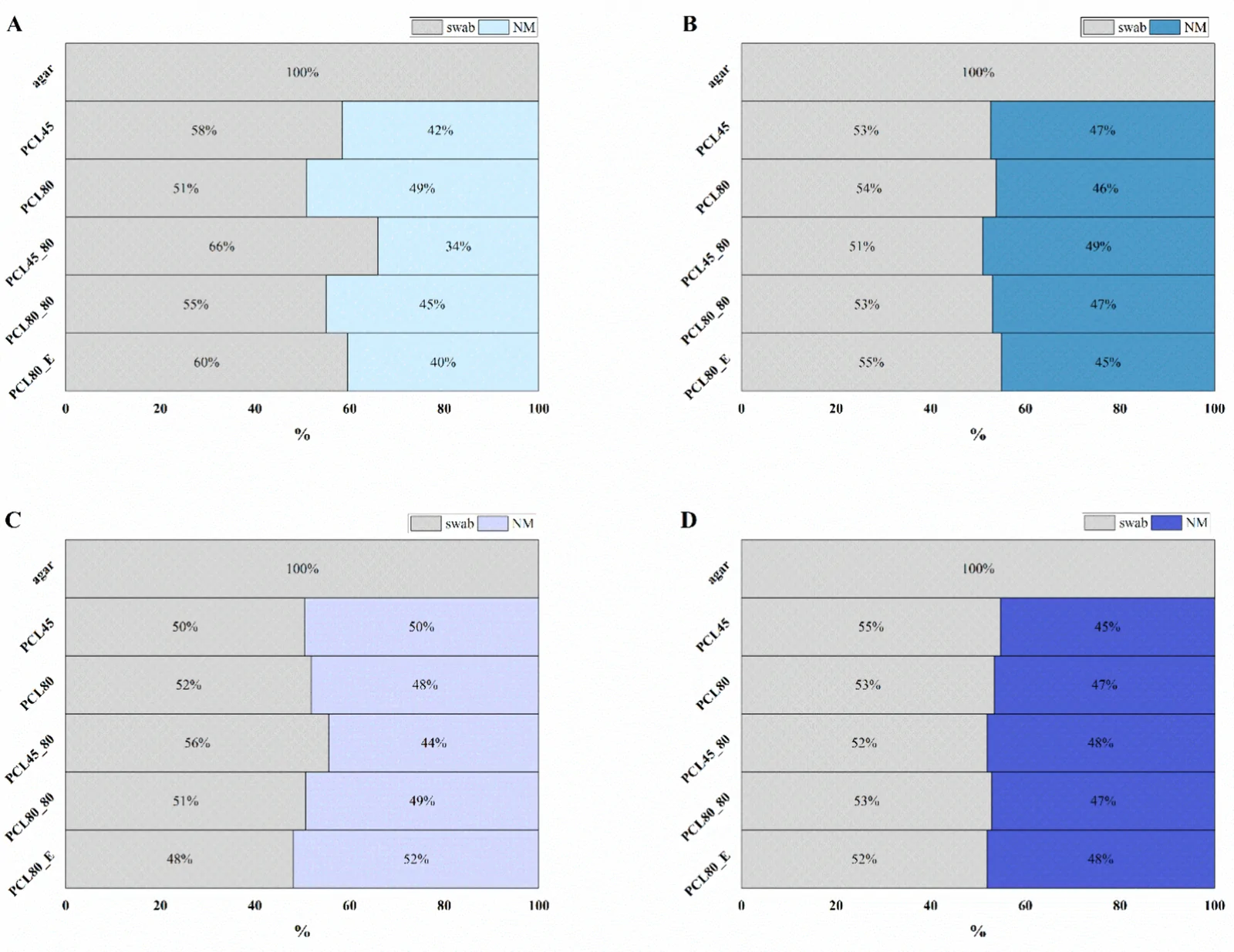
Ratio of agar-NM transfer for *S. cerevisiae* 9763 (A, B) and *S. cerevisiae* 8381 (C, D) for 2 h-long preincubation (A, C) and 72 h-long preincubation (B, D). Every NM transfer is relative to the total CFU count (CFU gained from agar on a swab and CFU gained from NM sonication).

Differences between individual materials appeared even more scarcely (ranging between 45.1 and 50.9 %) after longer colonization of surfaces. The mean CFU counts on MEA after 72 h of pre-incubation were 8.3 ± 0.1 log_10_(CFU/cm^2^) for *C. albicans* and 7.7 ± 0.2 log_10_(CFU/cm^2^) for *S. cerevisiae*, marking a difference of about 0.5 log_10_(CFU/cm^2^) between the species. Out of all tested NMs, PCL45_80 seemed to be able to attach the highest average number of CFU/cm^2^ (up to 50.9 % for *C. albicans* 10231, Figure 9B), although not always significantly. Except for *S. cerevisiae* 8381 (Figure 10D), PCL80 and PCL80_E were among the least suitable NMs for yeast removal, attaching only between 45.1 and 46.9 % of cells. Among the reasons causing this phenomenon can be the fiber diameter and the bigger secondary structures on the enzymatically degraded fiber surfaces. On the other hand, finer fibers of PCL45 and secondary structures created by spraying (PCL45_80) usually provided a slightly better environment for attachment, adding up to 2 % of total cell transfer. However, all these data indicate that H4: “NMs can transfer a significant number of yeast cells from agar plates.” cannot be confirmed. Nevertheless, SEM images show an almost uniform cover of all tested PCL NMs (see Figure 11), with visible ruptures most probably caused by the sample preparation and handling, and these findings could hint at a possible use for the preparation of small-scale active yeast surfaces not only on the PCL NMs.

**Figure 11:**
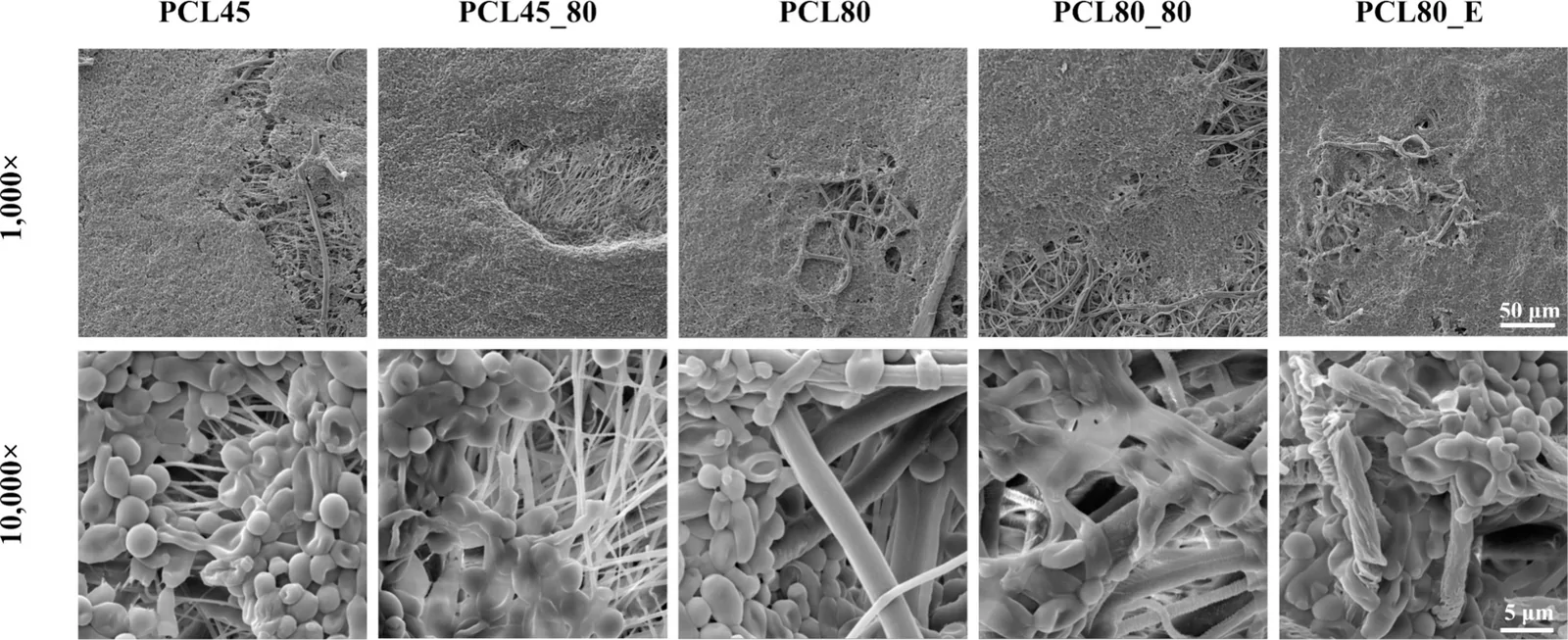
SEM images of transferred *C. albicans* 10231 cells on all tested materials after 72 h-long preincubation at 25 °C, shown at 1000× and 10,000× magnification. Most of the tears in the uniform layer of yeast cells were most probably caused by handling and manipulation.

### 3.3 Summary of PCL NMs–Yeast Interactions Results and Future Perspectives

PCL NMs and yeasts can interact on multiple levels, and the findings presented here can be applied in numerous fields of human activity, including medicine, biotechnology, the food industry, or water treatment. This study is the pilot one to systematically evaluate the interactions of both smooth-fibered NMs of two distinct mean fiber diameters and rough-fibered NMs created by two specialized post-electrospinning processes, i.e., polymer spraying and enzymatic degradation. It is important to note that yeasts behave differently on electrospun nanofibers than the smaller bacteria addressed in many other studies.^17,18,21,24,33^

Regarding filtration, all four tested NMs (smooth and rough fibers created by spraying) have been proven as effective filters, removing up to 100 % yeast cells from the filtered suspension with the maximal retention of 6.4 log_10_(CFU/mL). During the filtration, cells tend to cluster and, in subsequent cultivation, the rough-fibered PCL NMs do not seem to hinder their growth. Due to yeast size, the fiber diameter is not as relevant a factor as it is for bacteria; however, the lower retention rate has always been found in either PCL45 or PCL45_80 and could be caused by filter preparation (±20 g/m^2^). Heavier filters could be able to hold more yeast cells more easily, but this hypothesis would need further verification.

Like in the case of bacteria, yeast biofilm formation on PCL NMs cannot be generalized at either of the tested time points, be it adhesion, early biofilm phase, or mature biofilm phase. In some cases, the NMs seem to enhance or reduce the biofilm mass compared to the smooth PS surface, statistically significantly even; however, the change of about 1.5 log_10_(CFU/mL) or less may not be relevant enough for the desired application. Considering the material-specific properties, the possible errors caused by inoculation factors and data obtained for adhesion after 2 h incubation, it can be supposed that PCL NMs are harder to colonize by the tested strains of *Candida* and *Saccharomyces*. Furthermore, thinner fibers of PCL45(_80) could be used to help prevent yeast colonization because the CFU counts and thus even metabolic activity on them were often the lowest. On the other hand, yeast biofilms on PCL80_E were often among the most metabolically active, even though CFU counts were on par with other PCL80 NMs. This could mean that either the yeast cells react to the possible enzyme remaining among the fibers, or these particular secondary structures allow for a higher metabolism in biofilms, rendering them likely useful for technological purposes. SEM shows that yeasts, just like bacteria, tend to colonize the inner structures of the NMs, regardless of the secondary structures, if the pore size allows it.

Agar-NM transfer has been proven semi-effective, as only about half, usually less, of the yeast cells adherent to the agar surface could be transferred to the NMs. To obtain an efficiency similar to that in the case of bacteria, fibers with more attachment points would be needed. Nonetheless, the possible application of these specific PCL NMs (based on our data for 72 h preincubation, preferably PCL45 or PCL45_80) could be the creation of yeast active surfaces, because yeasts can almost uniformly cover the NM in only 1 h of contact.

## 4 Conclusions

PCL NMs are functional parts of many devices in medicine and industry. In this study, we have successfully managed to prepare a set of five PCL NMs with smooth and rough fibers and tested how their modified properties affect retention, growth, and cell transfer of two yeast species, namely, *C. albicans* and *S. cerevisiae*. Our obtained data indicated that (i) secondary structures on fiber surface do not influence the retention of the tested yeast cells, (ii) fiber diameter does not play a significant role in filtration due to yeast cell size, (iii) NMs-yeast interactions tend to differ mostly during the adhesion phase of biofilm formation, (iv) yeast biofilm is formed on all NMs, and its mass is strain-specific, (v) NMs with larger fiber diameter and/or enzymatic degradation (PCL80_E) appear to be the most suitable for high yeast colonization, and (vi) for yeast cell transfer, PCL45_80 seems to be the most promising support or scaffold material because it could attach the highest percentage of cells from the surface. These results are important for understanding biological interactions of secondary structures on nanofibers with microorganisms, namely, yeasts, which are often used in technological processes. Additionally, the presented findings can be beneficial for creating novel materials, with or without secondary structures, used to diminish or enhance yeast growth.

## Supplementary Material



## Data Availability

Raw data are available on Zenodo under DOI: 10.5281/zenodo.21507106.

## Notes

Images of FTIR analysis for PCL80 and PCL80_E, CV staining of biofilm formation between 2 and 96 h on PS for *C. albicans* 10231, *C. albicans* 90028, *S. cerevisiae* 9763 and *S. cerevisiae* 8381, additional SEM images of biofilm formation on tested materials at 24 h and 72 h (PDF)

## Abbreviations

BET: Brunauer–Emmett–Teller method
CFU: colony-forming units
CV: crystal violet
FTIR: Fourier transform Infrared
MEA: malt extract agar
MEB: malt extract broth
MTT: 3-(4,5-dimethylthiazol-2-yl)-2,5-diphenyl tetrazolium bromide
NM: nanofibrous material
OD: optical density
PA: polyamide
PAN: polyacrylonitrile
PBS: phosphate-buffered saline
PCL: polycaprolactone
PS: polystyrene
SEM: scanning electron microscopy
