# Supplementary material for: Influence of Fiber Diameter and Surface Structure of Polycaprolactone Nanofibrous Materials on Yeast Retention and Biofilm Formation
Source: ACS Omega. 2026 Oct 2;11(41):63692–706. doi: 10.1021/acsomega.6c07770 (PMC13688552; doi:10.1021/acsomega.6c07770)

Influence of fiber diameter and surface structure of polycaprolactone nanofibrous materials on yeast retention and biofilm formation

Marta Stindlova[1*], Eva Kuzelova Kostakova[2], Vera Jencova[2], Kristyna Havlickova[2], Vaclav Peroutka[1*], Kamila Zdenkova[1], Ema Chudobova[2], Jana Stepanova[3], David Lukas[2], Simona Peroutkova[1]

[1] University of Chemistry and Technology Prague, Faculty of Food and Biochemical Technology, Department of Biochemistry and Microbiology, Technicka 5, 166 28 Prague, Czech Republic

[2] Technical University of Liberec, Faculty of Science, Humanities and Education, Department of Chemistry, Studentska 1402/2, 461 17 Liberec, Czech Republic

[3] Technical University of Liberec, Institute for Nanomaterials, Advanced Technologies and Innovation, Department of Nanochemistry, Studentska 1402/2, 461 17 Liberec, Czech Republic

*corresponding authors: stindlom@vscht.cz ; peroutkv@vscht.cz

Figure S1: Comparison of infrared spectroscopy (FTIR spectra) for PCL80 (red lines) and PCL80_E (blue lines). Full spectra (A) and cropped spectra (B) with a visible difference in C-O stretching. The full PCL80_E spectrum shows no accumulation of typical degradation by-products, as evidenced by the absence of i) new broad absorption bands in the hydroxyl region (3200–3500 cm^{-1}) and ii) peak broadening in the carbonyl region (around 1720 cm^{-1}). These combined spectral features confirm that while the polymer matrix is actively undergoing scission, the resulting low-molecular-weight degradation fragments (bearing terminal -OH and -COOH groups) do not remain integrated within the residual bulk material and are effectively washed away into the surrounding environment.

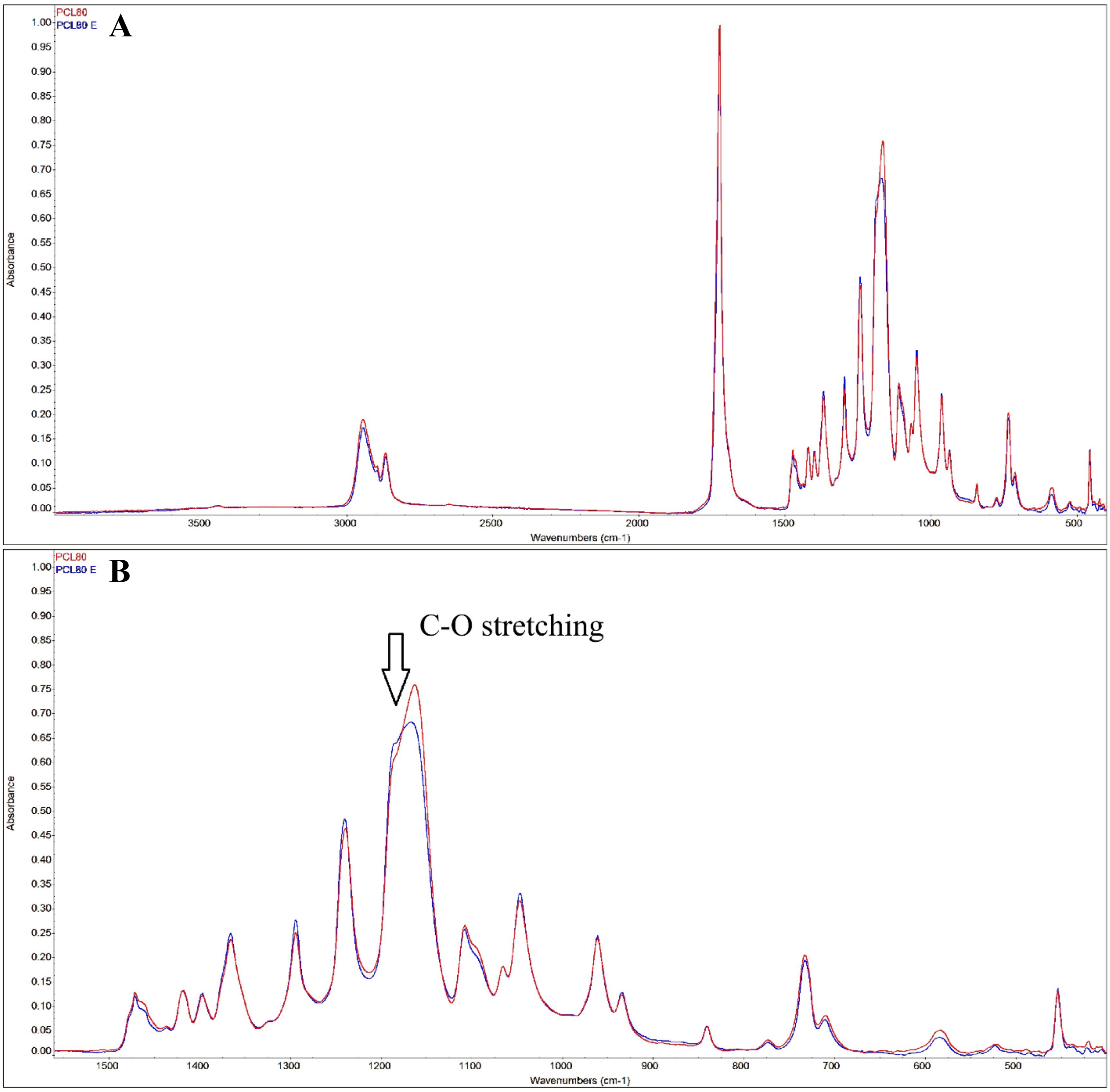

Figure S2: Crystal violet staining of different stages of biofilm formation on polystyrene for *C. albicans* ATCC 10231, *C. albicans* ATCC 90028, *S. cerevisiae* ATCC 9763 and *S. cerevisiae* CCM 8381. Magnification 400 x.

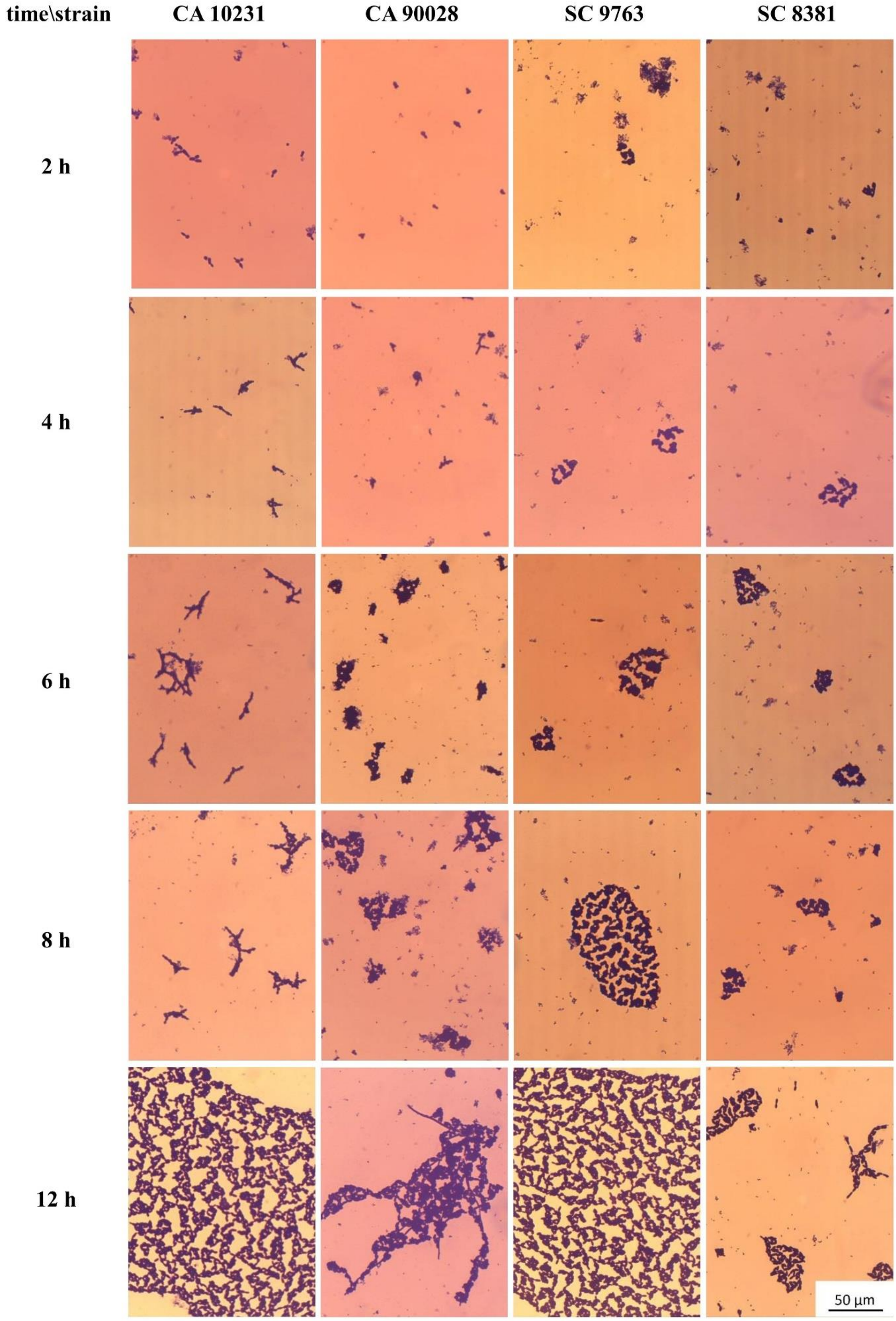

Figure S3: Crystal violet staining of different stages of biofilm formation on polystyrene for *C. albicans* ATCC 10231, *C. albicans* ATCC 90028, and *S. cerevisiae* ATCC 9763 and *S. cerevisiae* CCM 8381. Magnification 100 x.

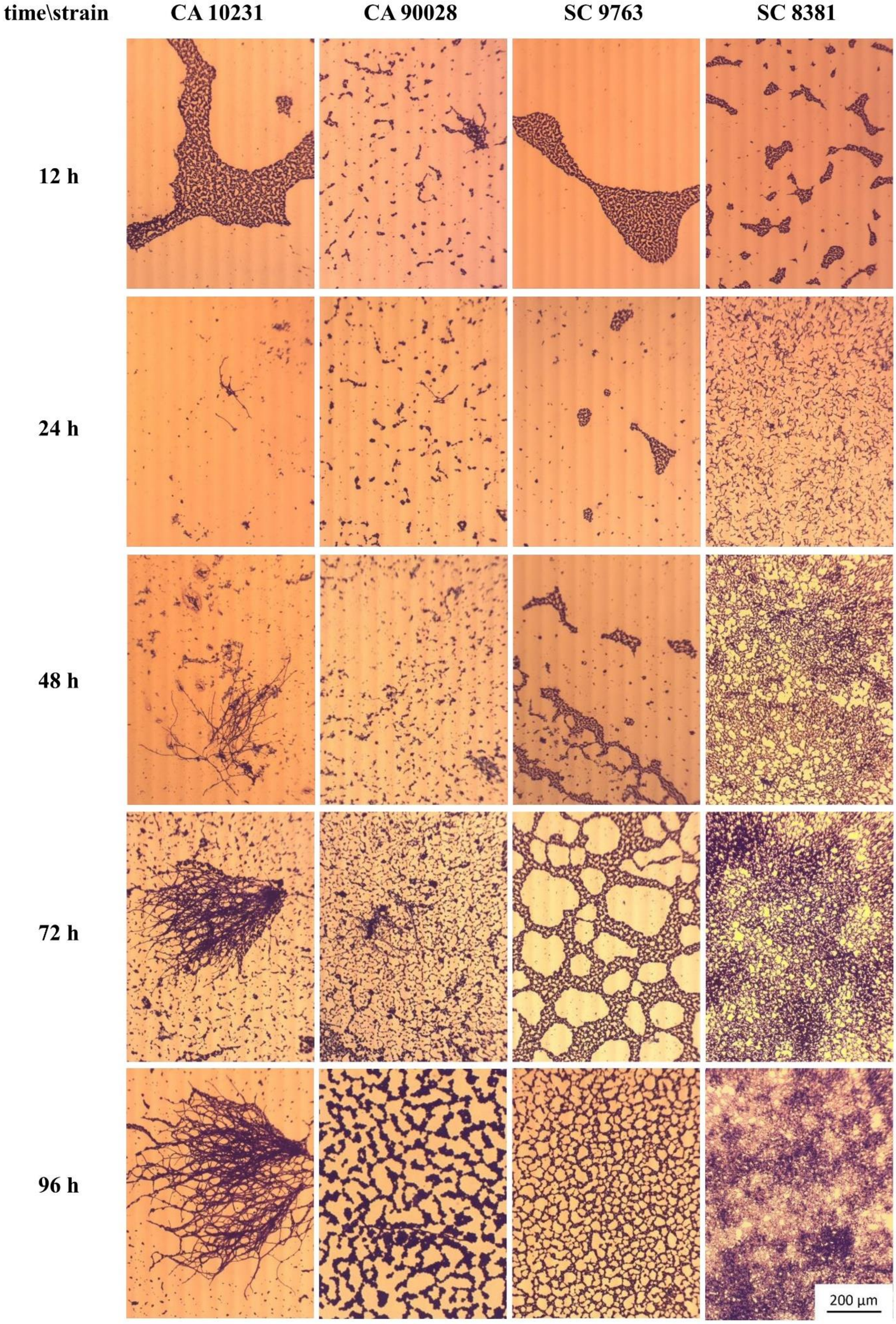

Figure S4: Relevant SEM images of *C. albicans* ATCC 90028, *S. cerevisiae* ATCC 9763, and *S. cerevisiae* CCM 8381 biofilms formed on PCL NMs at 24 h.

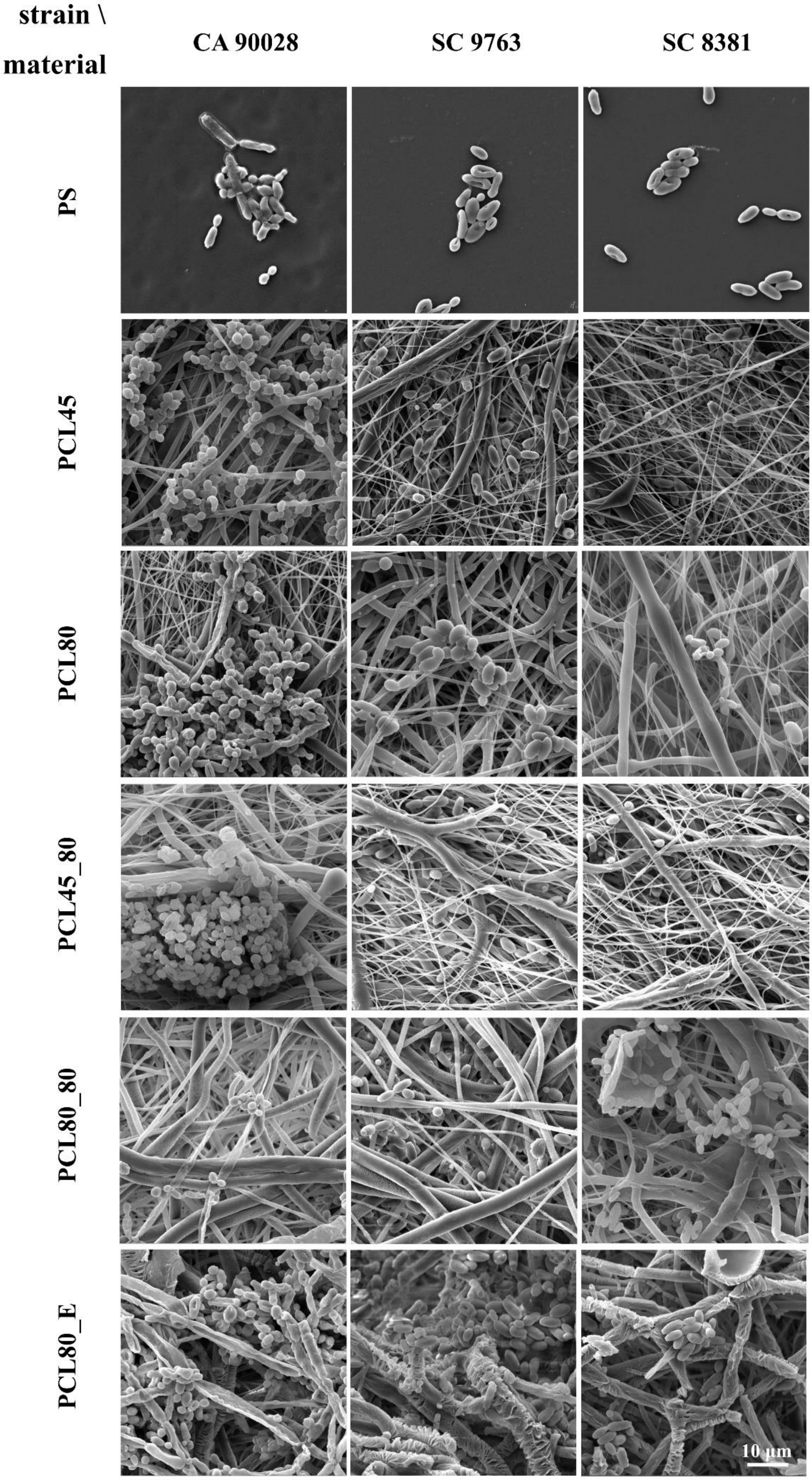

Figure S5: Relevant SEM images of *C. albicans* ATCC 90028, *S. cerevisiae* ATCC 9763, and CCM 8381 biofilms formed on PCL NMs at 72 h.

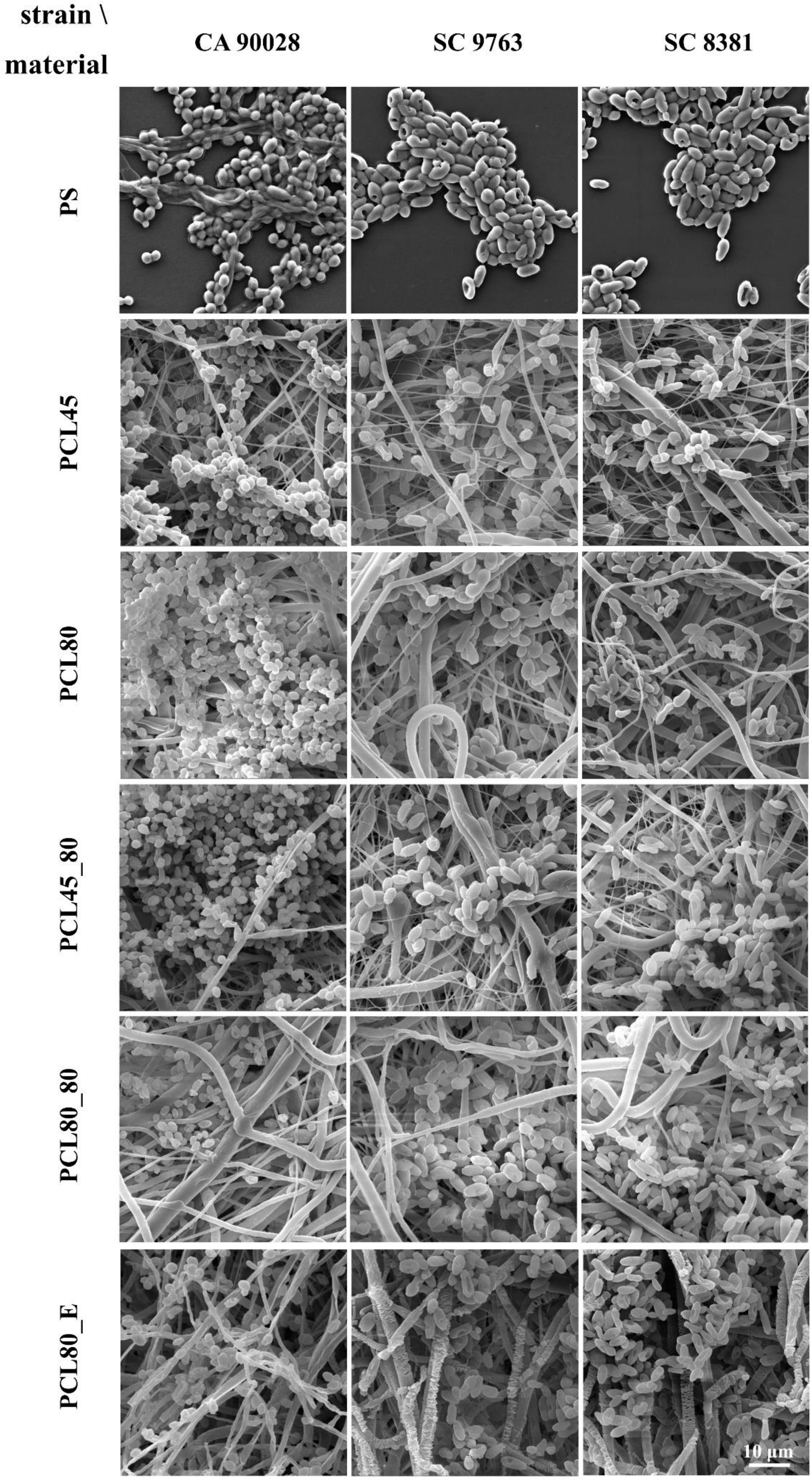

Supplement: Supplementary file 1 [file ao6c07770_si_001.pdf]
